# Design and implementation of corrosion-resistant multitasking cell stainers

**DOI:** 10.1371/journal.pone.0309334

**Published:** 2024-10-10

**Authors:** Chengsheng Liao, Zhen Gong, Run Fang, Leheng Li, Zhifan Gao, Min Mao, Libo Zeng

**Affiliations:** 1 School of Electronics and Electrical Engineering, Wuhan Textile University, Wuhan, Hubei, China; 2 Electronic Information School, Wuhan University, Wuhan, Hubei, China; 3 Hubei Province Engineering Research Center for Intelligent Micro-Nano Medical Equipment and Key Technologies, Wuhan, Hubei, China; National Taiwan Ocean University, TAIWAN

## Abstract

Compared with machine staining, traditional manual staining faces various problems, such as a low preparation success rate, low efficiency, and harm to the human body due to corrosive gases. Therefore, a stainer that is of low cost, has strong corrosion resistance, and is suitable for small-batch preparation should be developed. In this study, by choosing a rotary scheme as the structural basis, a reusable container cover and a master-slave manipulator cooperation scheme are developed, which greatly improve the space utilization rate. Through material selection and structural design, in the designed stainer, effective protection against strong acids with high volatility and permeability is realized, thereby eliminating the corrosion issue. Using the designed splitting-running algorithm for the dyeing procedure, simultaneous multistaining is realized, which significantly improves the staining efficiency. Compared with large-sized stainers, the cost of the proposed stainer is very low, which will help popularize early screening for cervical cancer in low- and middle-income countries.

## Introduction

Cervical cancer is the most common malignant tumor of the female reproductive system [1]. According to statistics, there were an estimated 604,127 cases of cervical cancer and 341,831 cervical cancer-related deaths worldwide in 2020 [2]. Currently, more than 85%of cervical cancer deaths occur in low- and middle-income countries [3]. An efficient screening method has an extremely important role and high significance in assisting clinical care and reducing cervical cancer mortality [4–6].

The main screening methods for cervical cancer are liquid-based cytology [7], virology-based test i.e. HPV-DNA test [8, 9], molecular marker test [10–12], colposcopy test [13], HPV-DNA combined with liquid-based cytology test [14, 15].

The ThinPrep cytologic test (TCT)based on liquid-based cytology are most likely to be popularized in low- and middle-income countries because of their simplicity and lower cost. The specific steps are as follows: collecting exfoliated cervical cells, preparing thin-layer cell smears, staining and fixation, and observing whether there are lesions under a microscope [16].

In low and middle income countries, the process of staining is mostly manual. Manual staining consists of dipping a prepared slide into a staining solution or dropping the solution onto the slide and then waiting for the staining time to arrive before taking the slide out and proceeding to the next staining step, with the process being repeated several times in a single staining process. manual staining is dominant but faces many problems: 1) The steps are homogenized, and prolonged fatigue may lead to confusion and errors. 2) Corrosive gases from dyeing are hazardous to health. 3) Manual dyeing is inefficient and requires long waiting time.

High-performance stainers are not widely available for the following main reasons: 1) High cost. The cost of the stainers is high, but the costs of other accessories and maintenance are even higher.

2) Poor applicability. In small hospitals and research laboratories, for cell staining, multibatch staining with a low workload for each batch is typical, and each staining process must be fast, while most commercially available stainers are designed for large-scale preparation.

3) Large volume and weight. Because the space in hospitals and laboratories is generally relatively limited, and most matrix stainers are relatively large, miniaturized and multifunctional stainers have become their first choice.

4) No corrosion resistance. Some dyeing processes require the use of strong acid dyes. Because volatile strong acids are highly permeable and corrosive, such dyeing processes can only be performed manually in a fume hood.

Cytological staining plays an important role in the whole process of cytological diagnosis; therefore, it is necessary to develop a corrosion-resistant and miniaturized multifunctional stainer suitable for use in small hospitals and laboratories.

## Materials and methods

### Corrosion-resistant stainers

To achieve corrosion resistance, in addition selecting suitable materials, various aspects must also be considered, such as the sealing solution, structural optimization, and protection treatment [17, 18]. The design and anticorrosion measures of several key structural components are described.

#### Design and anticorrosion measures of the working chamber

**Fig 1:**
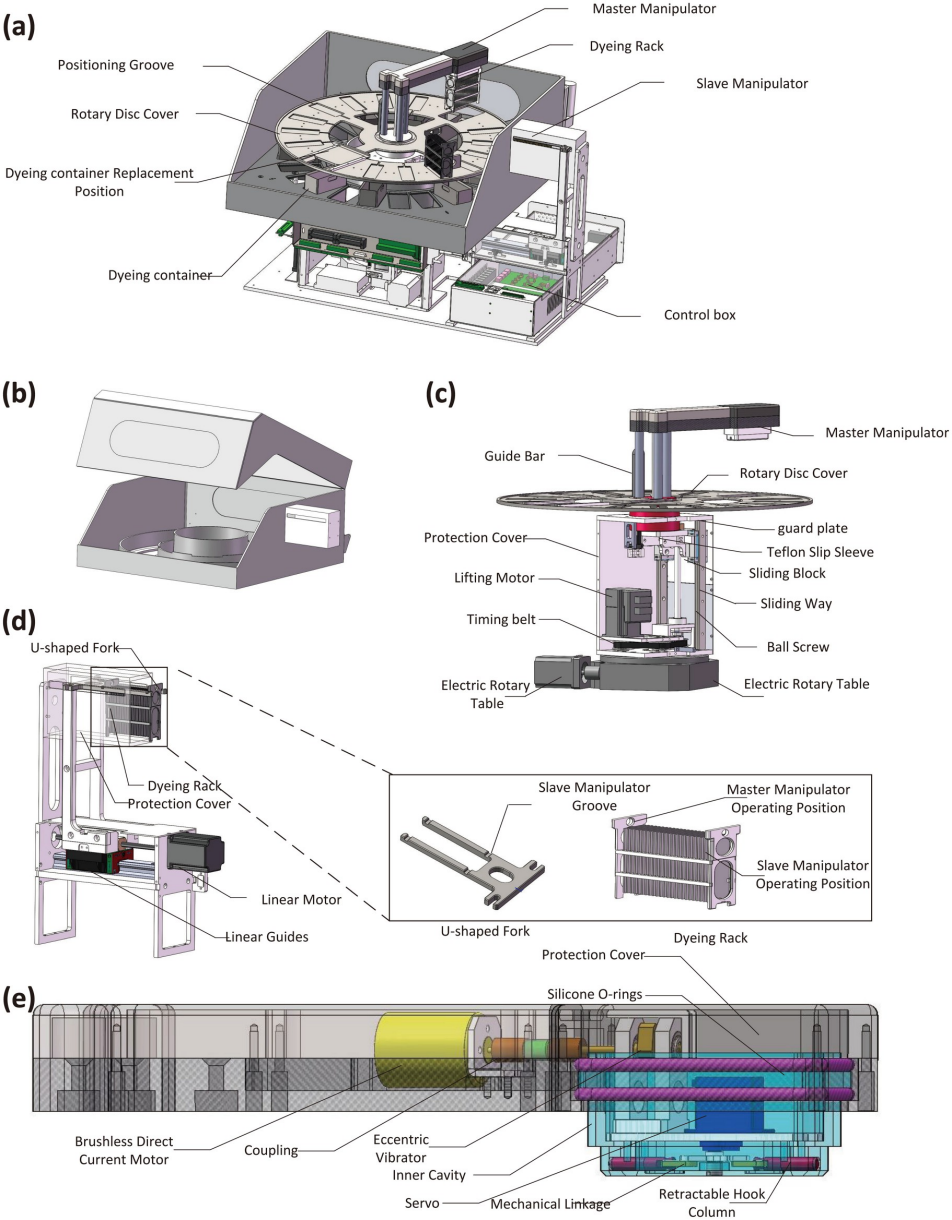
Cell stainers structure design. (a) Overall structure. (b) Working chamber. (c) Design of central motion module. (d) Design of slave manipulator and Dyeing rack. (e) Design of Master manipulator.

In this study, corrosion-resistant engineering plastics, such as acrylonitrile-butadiene-styrene (ABS), polytetrafluoroethylene (PTFE) and poly-vinyl chloride (PVC) [19–21], are used to form a working chamber as a container and for manipulator operation(Fig 1b). The working chamber is isolated from other external components, such as the control box, slave manipulator and liquid crystal display (LCD) screen, and the corrosive gas volatilized from the dye liquor can be controlled in the working chamber.

In the working chamber, there is also a rotary disc cover, as shown in the Fig 1a. The main function of the disc cover is to prevent the volatilization of the corrosive dye liquor. In contrast to the container arrangement in a matrix stainer, there is no need to design a separate cover or a complex cover opening and closing mechanism for each dyeing container; only one large disc cover that can cover all dyeing containers is sufficient since the disc cover can be rotated to any angle to ensure that all dyeing containers are covered. The disc cover can also be linked with the central motion module and has two degrees of freedom (DOFs), namely, lifting and rotation; consequently, there is no need to additionally design a cover opening and closing mechanism. In some dyeing processes, because the cells are not soaked in the dye solution, a positioning groove is designed on the top of the disc cover as a temporary storage and drying zone, which greatly improves the space utilization rate.

There is an openable cover on the working chamber for loading, unloading, and changing the dye liquor, and the chamber cover is kept closed during regular operation to prevent corrosive gas from leaking out of the working chamber. By using both the disc cover and chamber cover, the protection against dye liquor volatilization is doubled; thus, the concentration of corrosive gas in the working chamber may not be too high. With the filtered exhaust fan creating a slight negative pressure inside the working chamber, less corrosive gas can leak out from the working chamber.

#### Design and anticorrosion measures of the central motion module

The central motion module is a structure that assists in rotating and lifting the disc cover, and the structure of the central motion module is shown in detail in the Fig 1c. Key components, such as guide rails, motors, ball screws, and sliders, are installed inside a protective cover. The guide rail sliders and guide rods can move up and down under the drive of the lifting motor, and the electric rotary table at the bottom can rotate the disc cover. The connection part between the module and the working chamber is made of a corrosion-resistant and self-lubricating PTFE sliding sleeve [22, 23], and with the close cooperation of three guide rods, sealing surface is formed, which can move flexibly and maintain sealing. The sliding sleeve also closely cooperates with the guard plate to form sealing surface, which can rotate and move flexibly. The sliding sleeve is used to connect with the working chamber, and there is a hole in the working chamber that cooperates with the PTFE sliding sleeve to form a sealing surface to ensure that the corrosive gas in the working chamber cannot penetrate into the protective cover so that the corrosion resistance of the central motion module can be achieved.

#### Design and anticorrosion measures of the master-slave manipulator

The master manipulator can dip the dyeing rack into the container by rotating and lifting. However, to position the dyeing rack in the temporary storage zone, the manipulator must first be raised to a height exceeding that of the second layer of the dyeing rack and then must be lowered after being rotated to the predetermined position. Using this scheme, the lift and movement of the manipulator are long, which can increase the overall height of the stainer. Therefore, we add a slave manipulator to cooperate with the disc cover to transfer the dyeing rack to the designated position. The specific principle and method are as follows: On the dyeing rack, there is an operation hole for the master manipulator and a slot for the slave manipulator, as shown in the Fig 1d. When taking the dyeing rack from the manipulator, a U-shaped fork enters the dyeing rack and is aligned with the slot, allowing the dyeing rack to be transferred to the slot on the slave manipulator after lowering it. By reversing the above process, the dyeing rack can be suitably positioned. The lowering or lifting action of the dyeing rack can be realized by the master manipulator or by the lifting and lowering of the disc cover; that is, the operation of the dyeing rack can be switched between the master manipulator and the disc cover by using the slave manipulator as a relay.

The internal structure of the master manipulator is shown in the Fig 1e, and all components are installed inside a protective cover. The servo is connected to a hook column through a mechanical linkage and drives the hook column to perform reciprocating motion when it rotates. There are relative motion and sealing requirements between the hook column and the inner cavity. Therefore, the material of the inner cavity is the corrosion-resistant and self-lubricating PTFE, which can ensure the sealing of the inner cavity when the Retractable hook column performs reciprocating motion. The brushless direct current (DC) motor drives the eccentric vibrator to rotate at a high speed through coupling, which makes the inner cavity and internal components vibrate. When the dyeing rack is installed on the hook column, the hook column can drive the dyeing rack to vibrate, thereby draining the remaining liquid, which is the vibration waste draining function. Two silicone O-rings are used to provide a seal between the inner cavity and the protective cover. At the same time, the O-ring also has the functions of buffering and preventing corrosive gas penetration. Namely, it can keep the relative position between the inner cavity and the protective cover to maintain the positioning accuracy and provide buffering between the inner cavity and the protective cover to reduce the vibration amplitude transmitted to the protective cover and the main shaft. With these two seals, the penetration of corrosive gas into the inner cavity can be effectively prevented. For the slave manipulator, a protective cover with a groove is used(Fig 1d). The connection between the protective cover and working chamber is sealed, the U-shaped fork and the dyeing rack can move freely in the protective cover, and the liquid dripping from the dyeing rack is collected and recovered by the protective cover to prevent it from contaminating components, such as linear guides and linear motors. Because the exposed guide rod and hook column of the master-slave manipulators must be made of metals, titanium alloys with suitable mechanical and chemical properties are selected [24, 25].

### Multitask staining procedure

To meet the requirements of multibatch staining with a low workload for each batch, it is necessary to ensure the accurate control of the dyeing time and processes and to optimize the paths and algorithms to improve the efficiency. Therefore, efficient running algorithms for dyeing procedures are particularly important.

#### Running rules for dyeing procedures

Different dyeing methods have different dyeing protocols, and different dyeing protocols may differ in the type of dye solution, dye soaking time, and dyeing sequence for each dyeing step. In practice, using one dyeing rack as a unit to perform all steps of a dyeing type is called a dyeing procedure, which also can be understood as a thread or a task from a software engineering perspective. Therefore, this study discusses how to arrange each dyeing rack to execute all the steps in a reasonably orderly manner, i.e., the problem of thread running.

The easiest way is to use a single-task assembly line-style sequential algorithm to process only one dyeing rack at a time, and the next dyeing task can only be performed after all the dyeing steps for the previous dyeing rack are completed. This method is simple to implement and reliable in operation; However, the dyeing machine is idle most of the time waiting for the soaking time of a certain step of the dyeing solution to arrive before the next step of dyeing, resulting in low utilization rate of the apparatus. To improve the efficiency, the stainer can perform other tasks when it idles to wait for the dyeing to be completed, i.e., run multiple tasks at the same time, which is called a parallel running algorithm.

A parallel running scheme for the dyeing procedure can enable all dyeing racks to perform dyeing steps at the same time, but because the manipulator and some dyeing racks are shared, there will inevitably be a problem of resource competition during operation. To realize the parallel running of dyeing procedures, a general principle should be followed, that is, the soaking time in the dyeing step should not exceed the specified time, and excessive dyeing should be avoided, but the interval time between each step can be appropriately increased. Under this general rule, the following rules are proposed to resolve resource competition:

1) When the scheduled completion time for the current dyeing rack is reached, the dyeing container should be used by the next dyeing rack, and the first dyeing rack is placed in the temporary storage zone and waits for the dyeing container to be free;
2) When the scheduled completion time for the current dyeing rack is reached and the manipulator is not free, this dyeing rack is assigned the highest priority at this time, and this dyeing rack should be processed first when the manipulator is free;
3) When multiple dyeing racks in the temporary storage zone need to use the same dyeing container for the next dyeing process, the dyeing rack with the longest waiting time has the highest priority, and the other dyeing racks continue to wait;
4) When multiple dyeing racks are waiting for the manipulator at the same time, the dyeing rack with the longest waiting time has the highest priority;
5) Because a dyeing step with an extremely short soaking time (within 10 s) cannot be interrupted, its priority is the highest.

#### Splitting-running algorithm

Under the parallel running algorithm, various procedures and different steps could interfere and compete with each other. If there is no clear design, some procedures might not be executed for a long period of time, and some procedures might run over the scheduled time. Therefore, it is necessary to design an algorithm to solve this problem.

**Fig 2:**
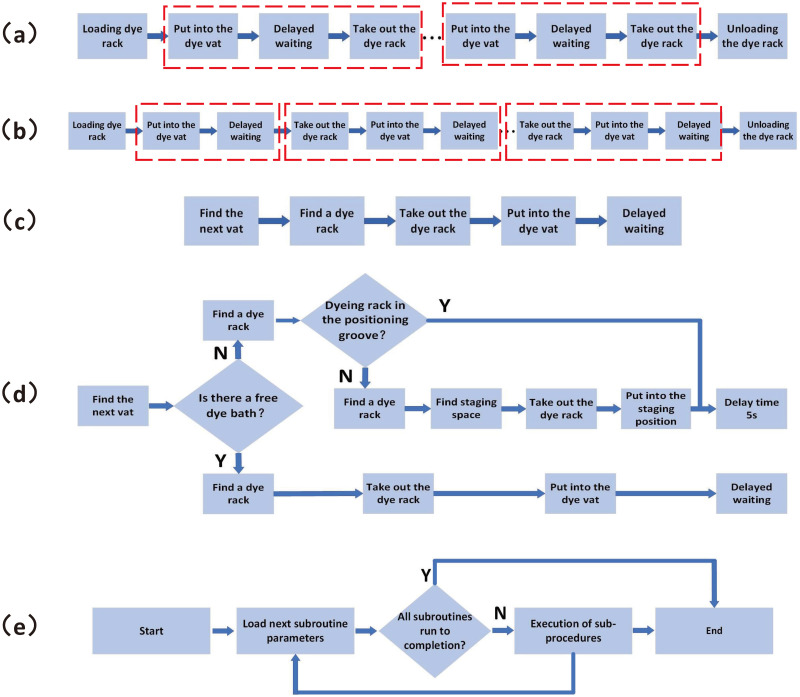
Dyeing protocol design. (a) Segmentation method according to actual workflow. (b) The “action-delay” beat division method. (c) Universal staining protocol. (d) Generic sub-protocols considering competition mechanisms. (e) Parent protocol running method.

The splitting-running algorithm splits the complex multistep staining procedure into multiple simple repeating subprocedures. According to the actual workflow, except for the operations of taking the dyeing rack at the beginning and unloading the dyeing rack after dyeing completion, all operations are cyclically executed, i.e., “putting the dyeing rack into the dyeing container, waiting, taking the dyeing rack out, and draining it”, which is a basic operation unit(Fig 2a). However, because this splitting method fails to make full use of the waiting time and is no different from the sequential algorithm in terms of programming, it is meaningless. If the basic operation unit of “action-waiting” is used and the operations of taking and draining in the previous dyeing step are moved to the next unit, then the basic operation unit can be changed to “taking out and draining the dyeing rack, placing the dyeing rack into the dyeing container, and waiting”(Fig 2b). In this basic operation unit, the stainer idles (waiting) after the dyeing rack is in the dyeing container and the manipulator is free. During the waiting time, the current tasks can be suspended or interrupted by other tasks. This splitting method is more efficient.

The subprocedure should also be universal, that is, regardless of the type of parent procedure or the number of parent procedures, the subprocedure can be run. When multiple procedures are running in parallel, the operations of finding the dyeing rack and finding the dyeing container should also be added to the subprocedure to obtain a general procedure(Fig 2c). The competition mechanism for dyeing containers should be added into the subprocedure, as shown in Fig 2d. If there is a free dyeing container, there is no competition. If there is no free dyeing container, the dyeing rack should be placed into the temporary storage zone for waiting. A scan is performed every 5 s until there is a free dyeing container. This mechanism resolves the problem of processing the dyeing rack under competition for dyeing containers.

According to this method, multiple dyeing procedures can be divided into same-type subprocedures, and the only differences lie in the input parameters, such as the type of dyeing solution and dyeing time. The parameter input is very easy to implement programmatically. At this point, the running of the parent procedure is simplified and organized into the following steps (Fig 2e): 1) recording the execution order of all subprocedures and the parameters of each subprocedure; 2) when running the procedure, loading and running the subprocedures in sequence; and 3) when all subprocedures have been loaded and run, the parent procedure is executed. In a multitasking operating system, a thread is established for each parent procedure, and multiple procedures are run in parallel. The priority of each thread in a multitasking operating system can be flexibly adjusted, which solves the problem of competition for manipulators.

#### Design of multitask staining procedure

**Fig 3:**
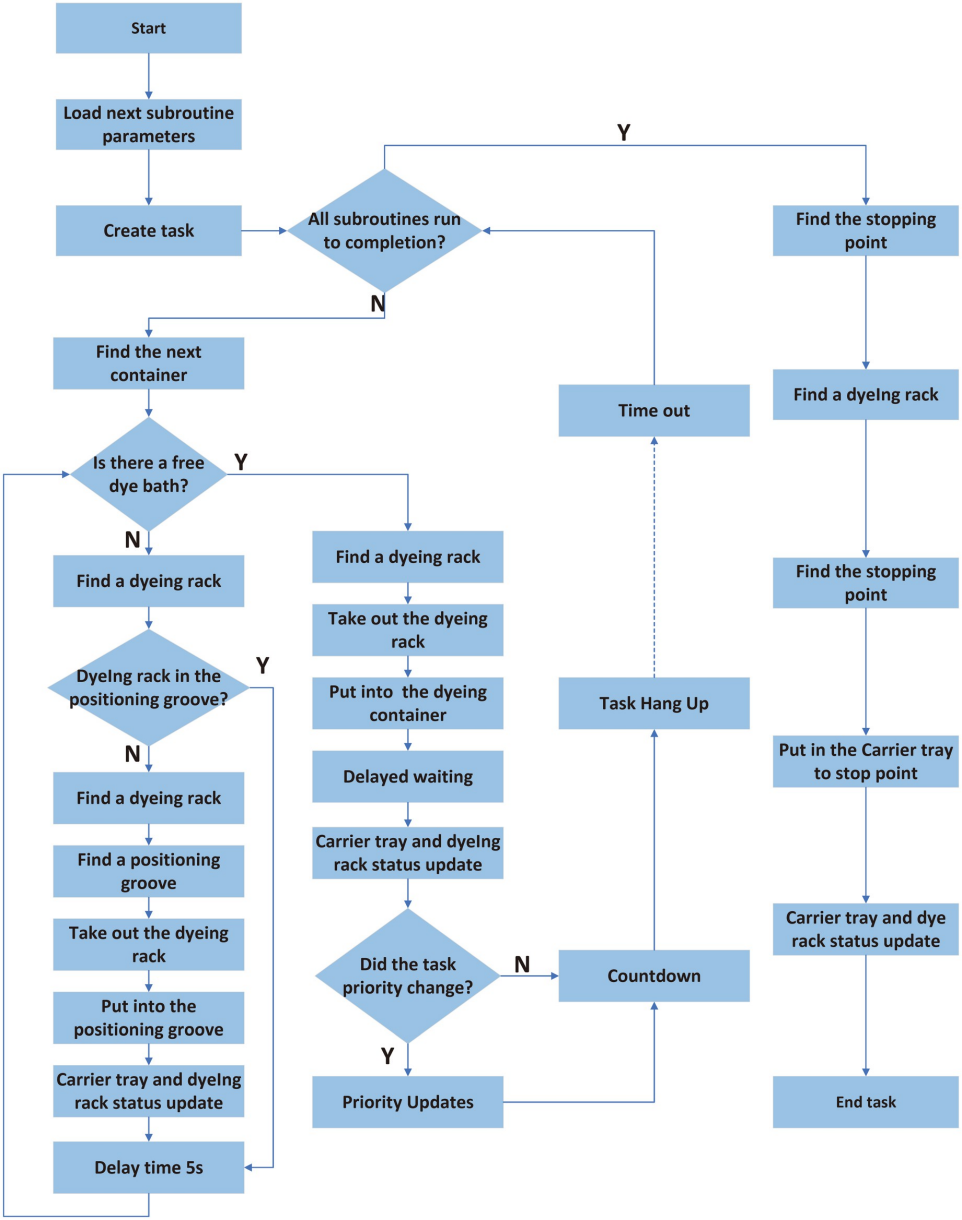
Dyeing scheduling main program flow chart.

The universal subprocedure considering the competition mechanism in Fig 2c is further designed and optimized into the main program of dyeing scheduling, as shown in Fig 3. After the system establishes the dyeing task and completes the initialization, the scheduler starts to execute. First, the dyeing container containing the corresponding dye liquor is searched for according to the dye liquor requirements revealed in the dyeing procedure information block. Then, the location of the dyeing rack is determined, and the dyeing rack is placed into the dyeing container to start dyeing. At this moment, the necessary dyeing time is obtained and stored in the time manager, and the countdown starts automatically backstage. At the same time, the task is suspended, and after the countdown is completed, the task is reactivated and enters the next dyeing subprocedure. Cycling is carried out until the dyeing is completed. Finally, the dyeing rack is placed on the free carrier disc, and the whole dyeing procedure is completed. Two competition mechanisms are also considered: for the dyeing container competition, if no suitable dyeing container is found, then the dyeing rack is placed in the temporary storage zone of the carrier disc, and a scan is performed every 5 s to check whether there is a suitable dyeing container; for the priority competition between the manipulator and dyeing container, the priority is determined once before the countdown, and necessary changes are made to update the priority according to the requirements.

## Results

**Fig 4:**
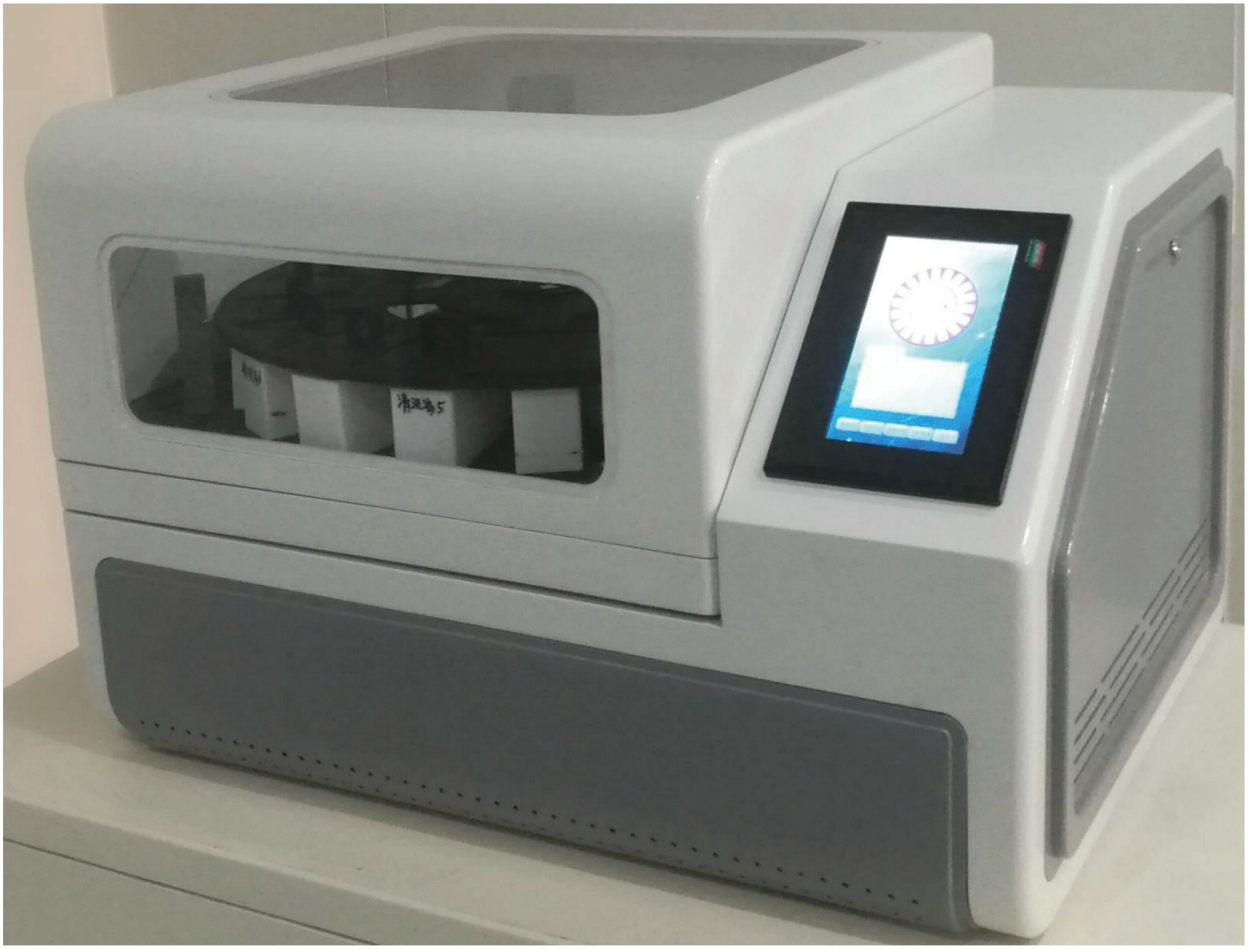
Image of the stainer.

Fig 4 shows the stainer prototype. The size of the stainer is 740 × 520 × 550 mm, the weight is 60 kg, and the height does not exceed 600 mm. The instrument is integrated with temperature control modules and other relevant modules below it. In addition, a liquid crystal touch screen is arranged in the upper right corner of the instrument to display relevant information on dyeing process, as well as to set various parameters of the cell stainers. Compared with commercially available stainers of the same type, it has a smaller volume and a higher space utilization rate.

The shell is made of ABS, which has good mechanical properties and corrosion resistance. Inside the working chamber, there are PTFE dyeing containers and structural parts. The master-slave manipulators are made of a titanium alloy, Bakelite and other materials, and sealing protection measures are taken. The test results for the stainer and each component show that the corrosion resistance meets the requirements. The total cost, including the costs of all spare parts and standard parts, machining fees, assembly fees, etc., is less than 20,000 yuan, and some parts are also processed by 3D printing instead of molding, which further reduces the cost.

## Discussion

### Positioning accuracy test

The center motion module has two DOFs: rotation and lifting. The lifting movement is used mainly to cooperate with the master-slave manipulators to take and place the dyeing rack, and there is a positioning allowance for both taking and placing the dyeing rack; that is, even if there is a slight deviation in the lifting height (±0.5 mm), an automatic correction can be performed to ensure correct loading and unloading. Therefore, the lifting DOF has low accuracy requirements. The stainer uses a C5 ball screw drive system, and the theoretical positioning accuracy is 23 mm, which can fully meet the requirements. The rotation is different and requires very high accuracy because when the master manipulator carries the dyeing rack into the dyeing container, the vertical position must be “aligned”; otherwise, mechanical damage could be caused if the dyeing rack cannot enter smoothly. In this positioning approach using the rotation angle, the angular error can be magnified at the end of the rotation radius, i.e., a small change in the angular displacement could cause a large change in the linear displacement at the end of the radius. Therefore, the angular positioning accuracy and transmission accuracy of the electric turntable directly determine the positioning accuracy of the master manipulator. After the system is built, the positioning accuracy and repeated positioning accuracy must be measured.

A Renishaw circular grating ruler is used to test the positioning accuracy of the master manipulator. The diameter of the carrier disc is 380 mm; therefore, a circular grating ruler with an outer diameter of 350 mm is selected to be concentrically installed on the carrier disc, and the reading head is connected to Renishaw’s special testing software, which can directly read the real-time coordinates and linear displacement. The outer circumference of the grating ruler, that is, the measurement length, is 3.14 × 350 = 1099 mm; the distance between the target positions is 200 mm; the number of targets is 5; and the positioning is repeated five times for each position. When the disc is rotated to and positioned at a target point *P*_*i*_, the actual anchor point is *P*_*ij*_, and the difference between Pi and *P*_*ij*_ is the positioning deviation *X*_*ij*_. The average positioning deviation ${\bar{X}}_{i}$ (1) and standard deviation (SD) *S*_*i*_ (2) of the point *P*_*i*_ can be obtained after taking n measurements to obtain the positioning accuracy (4) and repeated positioning accuracy (3) of the linear displacement of the manipulator.

$$\begin{array}{c} \bar{X_{i}}=\frac{1}{n}\sum_{j=1}^{n}X_{ij}=\frac{1}{n}\sum_{j=1}^{n}\left(P_{ij}-P_{i}\right) \end{array}$$ (1)

$$\begin{array}{c} S_{i}=\sqrt{\frac{1}{n-1}\sum_{j=1}^{n}{\left(X_{ij}-\bar{X_{i}}\right)}^{2}} \end{array}$$ (2)

$$\begin{array}{c} R=\text{max}(2S_{i}) \end{array}$$ (3)

$$\begin{array}{c} A=\text{max}(\bar{X_{i}}+2S_{i}) \end{array}$$ (4)

According to the rotational positioning accuracy of the electric turntable △*a* = 0.01^0^, the rotation radius of the worktable is R = 200 mm, and the theoretical positioning accuracy can be calculated as △*s* = △*a* × *R* = 0.01^0^ × *π*/180^0^ × 200*mm* = 0.035*mm*. The measured results are shown in Table 1, and the following results are calculated: the linear displacement positioning accuracy of the manipulator of the stainer is 0.11 mm, and the repeated positioning accuracy is 0.05 mm. For more information, see S1 Fig.

**Table 1:** Statistical table of the linear displacement positioning accuracy and repeated positioning accuracy of the manipulator.

Target Location *P*_*i*_/mm	Positioning deviation *X*_*ij*_ /*μ*m					Average Positioning deviation	Standard deviation	Positioning accuracy	Repeat Positioning accuracy
	1	2	3	4	5	${\bar{X}}_{i}$ /*μ*m	*S*_*i*_ /*μ*m	A /*μ*m	R/*μ*m
150	23.25	46.25	13.28	63.23	24.37	34.076	20.26		
350	22.27	67.23	28.26	56.28	34.32	41.672	19.22		
550	58.22	67.98	32.27	96.28	36.24	58.198	25.98	110.16	51.96
750	48.23	86.26	79.23	53.38	36.98	60.816	21.03		
950	77.23	36.82	48.26	67.36	34.24	52.782	18.90		

The calculated results and the measured results exhibit a slight discrepancy, primarily attributed to the imperfect fit between the motor gear and rack in the device, resulting in an elevation of transmission accuracy value. During installation of the motion module, even a minor gap within the mechanical structure can impact installation accuracy, leading to positioning errors. Furthermore, spindle rigidity directly influences torsional deformation resistance. A spindle with higher axial stiffness effectively mitigates deformation and thereby enhances positioning accuracy. Considering its impact on positioning accuracy, it is necessary for the size of the dye frame to be smaller than that of the dye cylinder in order to ensure alignment despite certain errors. The aforementioned two experimental results fall significantly below this threshold, thus meeting precision design requirements.

### Temperature control test

In some dyeing methods, there are requirements for the temperature during dyeing, so the temperature control effect of the dyeing machine has a great influence on the dyeing effect. The device utilizes a constant temperature control module to regulate the equipment’s temperature, which is capable of real-time temperature detection. When the current temperature falls below the set point, it activates the heater power supply for heating. Conversely, when the current temperature exceeds the set point, it deactivates the heater power supply to cease heating. To test the temperature control effect of the constant-temperature water bath system of the stainer, a constant-temperature control experiment is designed. The experimental scheme is as follows:The experimental scheme was as follows: different room temperatures, including 10°C, 20°C, and 30°C, were simulated using an air conditioner, and the target water temperatures were set at 35.0°C and 27.0°C (which are the most frequently used target temperatures during actual control). Once the temperature of the liquid was stable, the thermometer reading (with an accuracy of 0.1°C for a mercury thermometer) was recorded every minute for 12 minutes. The liquid temperature was monitored in real-time, and the temperature curve inside the container was plotted. A temperature control range of ±0.5°C was required. The closer the temperature curve fluctuated to the target temperature, and the smaller the fluctuation amplitude, the better the temperature control effect. The collected data was tabulated as shown in Table 2. For more information, see S2 Fig.

**Table 2:** Data record table of temperature stability experimental.

Time/min		1	2	3	4	5	6	7	8	9	10	11	12
Target temp	Room temp	Liquid temp/°C											
27°C	10°C	27.0	26.8	26.9	27.1	26.6	26.8	26.9	27.3	27.2	27	26.7	26.8
	10°C	27.3	26.8	27.1	26.8	27.3	26.6	27.2	26.8	27.3	26.8	27.2	27.3
	10°C	26.8	27.0	26.9	27.1	27.1	26.9	27.4	27.2	26.7	26.8	27.0	27.2
	20°C	27.3	26.8	26.6	27.2	26.6	27.3	26.7	26.9	27.1	26.9	27.0	27.3
	20°C	26.7	27.3	26.8	27.0	27.2	27.1	26.7	26.8	27.2	27.0	26.7	27.0
	20°C	27.1	27.3	27.0	26.7	26.9	27.1	26.7	27.0	27.4	27.4	27.1	26.7
	30°C	26.8	26.7	26.7	27.1	27.0	27.2	27.3	26.9	27.0	27.1	27.0	27.2
	30°C	26.8	27.2	27.2	26.8	27.0	27.0	26.7	27.3	26.8	27.4	26.7	26.8
	30°C	27.3	27.0	26.7	27.3	27.3	26.8	26.8	27.2	27.1	26.8	26.7	26.6
35°C	10°C	35.2	35.3	34.9	35.0	34.8	34.6	35.2	35.0	34.9	35.1	34.8	34.9
	10°C	35.3	35.2	35.1	34.8	34.8	35.0	34.9	35.0	35.0	34.9	34.7	34.7
	10°C	34.9	34.8	35.1	34.7	35.2	34.6	35.1	34.8	35.2	35.4	34.6	35.2
	20°C	35.0	34.8	34.7	34.8	34.6	35.2	34.8	35.2	34.7	34.8	35.3	35.1
	20°C	35.3	34.7	35.2	35.3	35.3	34.8	35.2	35.2	34.8	35.2	35.3	35.0
	20°C	35.2	35.4	35.2	35.3	34.8	34.7	35.1	34.8	34.6	34.6	34.7	34.8
	30°C	34.8	35.4	35.2	34.7	34.7	35.2	35.4	35.0	35.2	35.1	34.8	35.0
	30°C	35.3	35.3	35.0	34.9	34.7	34.7	34.7	35.4	34.7	35.0	35.2	35.0
	30°C	35.3	35.0	34.9	35.1	34.7	34.8	34.9	35.1	34.6	34.7	34.7	35.1

**Fig 5:**
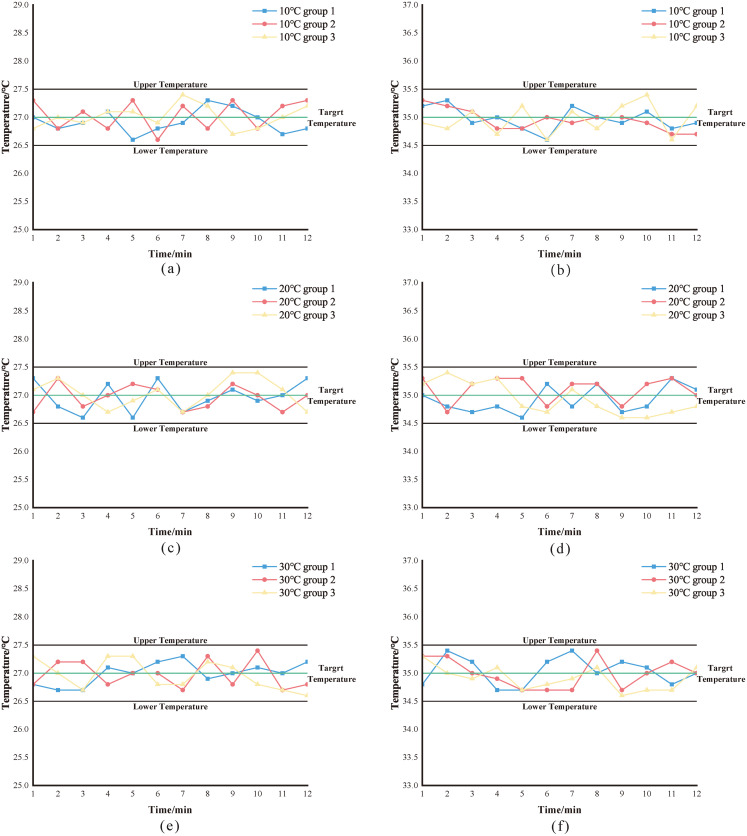
Temperature effect test line graph (a), (c), (e) Temperature fluctuation curve at a set temperature of 27°C (b), (d), (f) Temperature fluctuation curve at a set temperature of 35°C.

Plot the data in the Table 2 as a curve as shown in Fig 5. it was calculated that for a target temperature of 27°C, the maximum temperature (Tmax) was 27.4°C, the minimum temperature (Tmin) was 26.6°C, the average temperature was 26.987°C, and the standard deviation was 0.230. For a target temperature of 35°C, Tmax was 35.4°C, Tmin was 34.6°C, the average temperature was 34.977°C, and the standard deviation was 0.237.

The design of this temperature-controlled water bath system achieved the control requirement of ±0.5°C with small fluctuations. Therefore, the design scheme of this temperature control system is feasible.

### Dyeing efficiency test

In order to verify the efficiency improvement of multitasking staining, a comparative experiment on staining efficiency was conducted. Using the example of the Papanicolaou staining method, the time efficiency improvement of staining was first measured. Specifically, the staining machine was first allowed to perform single-task Papanicolaou staining and the overall staining time was measured and recorded. Then, the staining machine was used to perform multitasking Papanicolaou staining with multiple dyeing racks, and the time required for staining with different numbers of tasks performed simultaneously was measured. Subsequently, the average staining time per slide for both single-task and multitasking staining was calculated and compared.

**Fig 6:**
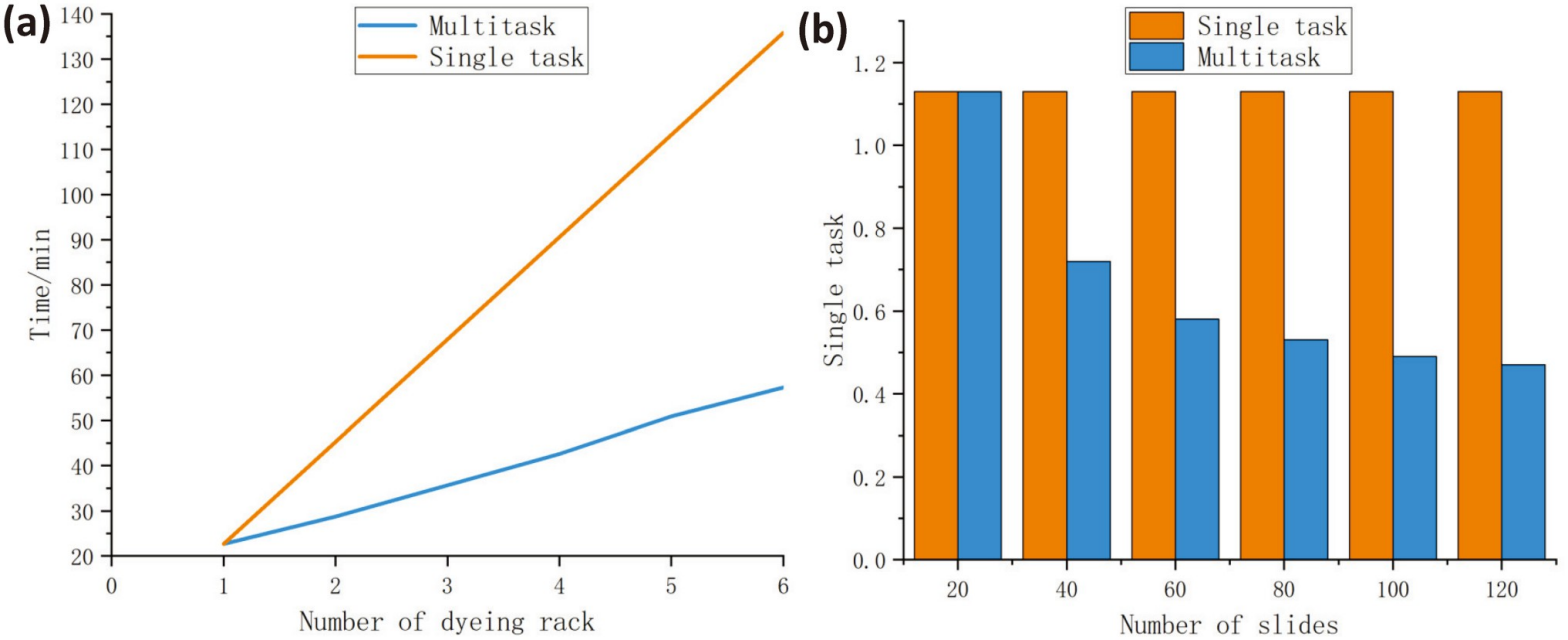
Dyeing machine effect test. (a) Comparison Chart of Single Task Pap Staining Effect. (b) Comparison Chart of Multitasking Forgan Staining Effect.

This study used a rack that can hold 20 slides for the experiment, and calculated the average staining time per slide using single-task and multitasking staining. The specific data is shown in the Table 3. Plot the data in the Table 3 as a curve as shown in Fig 6a. For more information, see S3 Fig.

**Table 3:** Time statistics table for single-task and multi-task staining.

Single-task	Number of slides	20	40	60	80	100	120
	Time/min	22.64	45.28	67.92	90.56	113.20	135.84
	Average time/min	1.13	1.13	1.13	1.13	1.13	1.13
Multitasking	Number of slides	20	40	60	80	100	120
	Time/min	22.64	28.72	34.64	42.58	49.39	56.46
	Average time/min	1.13	0.72	0.58	0.53	0.49	0.47

From the Fig 6a, it can be seen that when only one rack is used, the time consumption of single-task and multitasking staining procedures is the same. However, with the increase of rack number, the time consumption of multitasking staining is significantly lower than that of single-task staining. Even when the number of racks up to three, the time consumption of single-task staining is twice that of multitasking staining.

Based on the Fig 6b, it can be observed that the average time taken to stain a single slide significantly decreases as the number of staining racks used in multi-task staining increases, exhibiting a significant advantage over single-task staining. Especially when the number of racks exceeds three, i.e., the number of slides exceeds 60, the average staining time per slide in multi-task staining is significantly lower than that of single-task staining, by even up to half the time. Therefore, the use of this equipment can significantly improve the efficiency of dyeing.

### Dyeing effect test

Dyeing effect is the most important standard to examine the quality of the dyeing machine. The method of this test is as follows: first use this equipment to carry out single-task pasteurization staining, then carry out single-task new composite staining, and finally carry out multitasking staining, the multitasking specific steps are as follows: use four dyeing racks, dyeing racks 1 and 3 are set for pasteurization staining, and dyeing racks 2 and 4 are set for new composite staining, which are respectively added to the staining machine and run automatically at the same time. After staining was completed, the staining effect was observed using a cell scanner.

**Fig 7:**
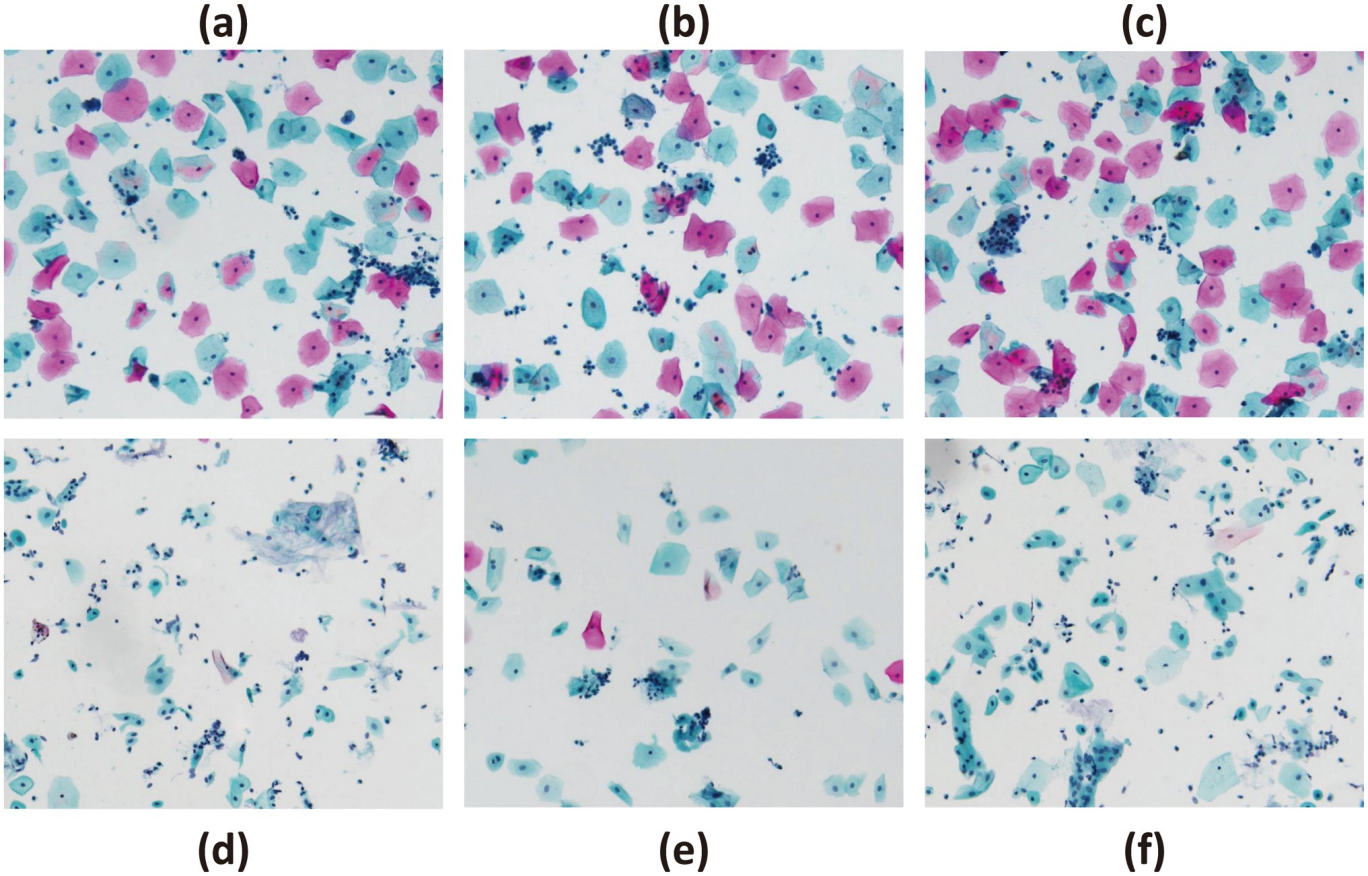
Cell staining effect. (a) Effect of single-task pasteurization staining. (b) Effect of multitasking pasteurization staining(Dyeing rack 1). (c) Effect of multitasking pasteurization staining(Dyeing rack 3). (d) Effect of multitasking new composite dyeing. (e) Effect of multitasking new composite dyeing(Dyeing rack 2). (f) Effect of single-tasking new composite dyeing(Dyeing rack 4).

As shown in the Fig 7 for the staining effect of the above multiple tasks, it can be seen that the staining effect is clear in the figure, and the contrast between the cytoplasm and the nucleus of the cell is sharp, which can be distinguished by the naked eye, which indicates that the staining quality of the staining machine is good. Through the same kind of staining mode under different task mode comparison can be seen under multi-task mode staining effect and single task no difference, indicating that the staining operation is accurate, reliable operation.

To eliminate the subjectivity of visual observation and the variability of experiments, the aforementioned dyeing steps were repeated. The data from the stained slides were processed using automatic scanning imaging equipment to assess the quality of dyeing. This device is capable of analyzing the integrated optical density (IOD) of cell slides, with higher values indicating better staining quality. IOD can be defined as the sum of the pixel optical density values within the measured structure range. It reflects the comprehensive variation of optical density and area of the measured structure. IOD is directly proportional to the substance’s mass, and its value reflects the relative content of the substance. According to device analysis, an IOD value of 10 or above is required for satisfactory results; any value below 10 indicates poor dyeing effect. Statistical analysis of IOD values is presented in the Table 4. For more information, see S4 Fig.

**Table 4:** Test results of IOD values under different staining modes.

Type of Staining	Type of task	Number of samples	Mean value of IOD	Standard deviation	Number of samples with IOD <10
Pasteurization staining	Artificial	100	19.04	0.2217	1
	Single-task	100	19.42	0.1601	0
	Multitask	100	18.59	0.1489	0
New composite staining	Artificial	100	18.97	0.2152	2
	Single-task	100	17.93	0.1452	0
	Multitask	100	18.29	0.1536	0

As illustrated in the table, human errors during the staining process inevitably result in some cell slides being improperly stained, leading to a higher standard deviation of IOD in manual staining. In contrast, both single-task and multi-task modes in automatic dyeing exhibit smaller IOD standard deviations compared to manual dyeing, indicating improved dyeing stability of the device. Furthermore, due to the precision control system, no samples with an IOD below 10 were detected in repeated experiments, highlighting that the device’s dyeing effect meets practical application requirements.

## Conclusions

In this study, we designed a corrosion-resistant multitasking stainer for cell staining during early cervical cancer screening.

In the design of the mechanical structure, we focused on better space utilization and corrosion resistance. We designed the rotating structure of the dyeing cylinder cover and the master-slave arm structure to reduce the volume of the equipment; we designed the working chamber to prevent the volatilization of corrosive gases, and the anti-corrosion strategy of the master-slave arm to reduce the corrosion. Through these designs, the space utilization and corrosion resistance of this equipment have been greatly improved.

In the design of software program, we fully utilize the time of dyeing liquid immersion by splitting the running algorithm, and make the dyeing protocol more reasonable by effective competition mechanism, which greatly improves the efficiency of dyeing without decreasing the dyeing effect, through the actual test, when the number of dyeing racks is more than 3 i.e., the number of slides is more than 60, the average dyeing time of single slide in multi-task dyeing is less than double of single-task dyeing.

In terms of cost, through the use of corrosion-resistant materials in key parts and the combination of 3D printing technology, the cost of the equipment has been greatly reduced.

Through the positioning accuracy test, temperature control effect test, dyeing efficiency test and actual dyeing effect test of the finished equipment, this equipment can meet the actual use requirements of hospitals.

## Supporting information

S1 Fig Positioning accuracy data. (PDF)

S2 Fig Temperature control data. (PDF)

S3 Fig Dyeing time data. (PDF)

S4 Fig Cell slide IOD scan data. (PDF)
