# Supplementary material for: Design and implementation of corrosion-resistant multitasking cell stainers
Source: PLoS One. 2024 Oct 10;19(10):e0309334. doi: 10.1371/journal.pone.0309334 (PMC11466404; doi:10.1371/journal.pone.0309334)
Supplement: S1 Fig — (PDF) [file pone.0309334.s001.pdf]

Positioning accuracy data							
	Date	Time	Target Location/mm	Positioning deviation/μm	Abnormal condition	Recorder	Reviewer
1	6/2/2023	9:13 a.m	150	23.25	None	Leheng Li、Ming Mao	Run Fang
2	6/2/2023	9:22 a.m	150	46.25	None	Leheng Li、Ming Mao	Run Fang
3	6/2/2023	9:32 a.m	150	13.28	None	Leheng Li、Ming Mao	Run Fang
4	6/2/2023	9:41 a.m	150	63.23	None	Leheng Li、Ming Mao	Run Fang
5	6/2/2023	9:50 a.m	150	24.37	None	Leheng Li、Ming Mao	Run Fang
6	6/2/2023	10:09 a.m	350	22.27	None	Leheng Li、Ming Mao	Run Fang
7	6/2/2023	10:20 a.m	350	67.23	None	Leheng Li、Ming Mao	Run Fang
8	6/2/2023	10.31 a.m	350	28.26	None	Leheng Li、Ming Mao	Run Fang
9	6/2/2023	10.41 a.m	350	56.28	None	Leheng Li、Ming Mao	Run Fang
10	6/2/2023	10:49 a.m	350	34.32	None	Leheng Li、Ming Mao	Run Fang
11	6/2/2023	3:11 p.m	550	58.22	None	Leheng Li、Ming Mao	Run Fang
12	6/2/2023	3:21 p.m	550	67.98	None	Leheng Li、Ming Mao	Run Fang
13	6/2/2023	3:30 p.m	550	32.27	None	Leheng Li、Ming Mao	Run Fang
14	6/2/2023	3:42 p.m	550	96.28	None	Leheng Li、Ming Mao	Run Fang
15	6/2/2023	3:53 p.m	550	36.24	None	Leheng Li、Ming Mao	Run Fang
16	7/2/2023	9:15 a.m	750	48.23	None	Leheng Li、Ming Mao	Run Fang
17	7/2/2023	9:27 a.m	750	86.26	None	Leheng Li、Ming Mao	Run Fang
18	7/2/2023	9:36 a.m	750	79.23	None	Leheng Li、Ming Mao	Run Fang
19	7/2/2023	9:46 a.m	750	53.38	None	Leheng Li、Ming Mao	Run Fang
20	7/2/2023	9:58 a.m	750	36.98	None	Leheng Li、Ming Mao	Run Fang
21	7/2/2023	10:17 a.m	950	77.23	None	Leheng Li、Ming Mao	Run Fang
22	7/2/2023	10:28 a.m	950	36.82	None	Leheng Li、Ming Mao	Run Fang
23	7/2/2023	10:27 a.m	950	48.26	None	Leheng Li、Ming Mao	Run Fang
24	7/2/2023	10:39 a.m	950	67.36	None	Leheng Li、Ming Mao	Run Fang
25	7/2/2023	10:36 a.m	950	34.24	None	Leheng Li、Ming Mao	Run Fang
