# Supplementary material for: Design and implementation of corrosion-resistant multitasking cell stainers
Source: PLoS One. 2024 Oct 10;19(10):e0309334. doi: 10.1371/journal.pone.0309334 (PMC11466404; doi:10.1371/journal.pone.0309334)
Supplement: S2 Fig — (PDF) [file pone.0309334.s002.pdf]

Temperature control data																						
Numbered	Date	Time	Target temp/℃	Room temp/℃	Recording time/min															Abnormal condition	Recorder	Reviewer
					1	2	3	4	5	6	7	8	9	10	11	12	13	14	15			
					Real-time temperature/℃																	
1	13/2/2023	9:23 a.m	27	10	27	26.8	26.9	27.1	26.6	26.8	26.9	27.3	27.2	27	26.7	26.8	26.8	26.7	26.8	None	Leheng Li、Ming Mao	Chengsheng Liao
2	14/2/2023	3:31 p.m	35	10	35.2	35.3	34.9	35	34.8	34.6	35.2	35	34.9	35.1	34.8	34.9	34.6	35.1	35.2	None	Leheng Li、Ming Mao	Chengsheng Liao
3	14/5/2024	9:19 a.m	27	10	27.3	26.8	27.1	26.8	27.3	26.6	27.2	26.8	27.3	26.8	27.2	27.3	27.2	27	27	None	Zhen Gong	Chengsheng Liao
4	14/5/2024	9:57 a.m	27	10	26.8	27	26.9	27.1	27.1	26.9	27.4	27.2	26.7	26.8	27	27.2	27.3	27	27.2	None	Zhen Gong	Chengsheng Liao
5	14/5/2024	3:21 p.m	27	20	27.3	26.8	26.6	27.2	26.6	27.3	26.7	26.9	27.1	26.9	27	27.3	27.2	26.7	27.4	None	Zhen Gong	Chengsheng Liao
6	14/5/2024	4:06 p.m	27	20	26.7	27.3	26.8	27	27.2	27.1	26.7	26.8	27.2	27	26.7	27	27.2	26.6	27	None	Zhen Gong	Chengsheng Liao
7	15/5/2024	9:22 a.m	27	20	27.1	27.3	27	26.7	26.9	27.1	26.7	27	27.4	27.4	27.1	26.7	27.3	26.8	27.4	None	Zhen Gong	Chengsheng Liao
8	15/5/2024	9:59 a.m	27	30	26.8	26.7	26.7	27.1	27	27.2	27.3	26.9	27	27.1	27	27.2	27	27.2	26.9	None	Zhen Gong	Chengsheng Liao
9	15/5/2024	3:18 p.m	27	30	26.8	27.2	27.2	26.8	27	27	26.7	27.3	26.8	27.4	26.7	26.8	26.7	26.6	26.7	None	Zhen Gong	Chengsheng Liao
10	15/5/2024	3.57 p.m	27	30	27.3	27	26.7	27.3	27.3	26.8	26.8	27.2	27.1	26.8	26.7	26.6	26.7	26.6	26.6	None	Zhen Gong	Chengsheng Liao
11	16/5/2024	9:19 a.m	35	10	35.3	35.2	35.1	34.8	34.8	35	34.9	35	35	34.9	34.7	34.7	34.8	35.2	34.9	None	Zhen Gong	Chengsheng Liao
12	16/5/2024	9:59 a.m	35	10	34.9	34.8	35.1	34.7	35.2	34.6	35.1	34.8	35.2	35.4	34.6	35.2	35.1	35.3	35.1	None	Zhen Gong	Chengsheng Liao
13	16/5/2024	3:20 p.m	35	20	35	34.8	34.7	34.8	34.6	35.2	34.8	35.2	34.7	34.8	35.3	35.1	34.6	34.7	35	None	Zhen Gong	Chengsheng Liao
14	16/5/2024	4:00 p.m	35	20	35.3	34.7	35.2	35.3	35.3	34.8	35.2	35.2	34.8	35.2	35.3	35	34.9	35.2	35.4	None	Zhen Gong	Chengsheng Liao
15	17/5/2024	9:25 a.m	35	20	35.2	35.4	35.2	35.3	34.8	34.7	35.1	34.8	34.6	34.6	34.7	34.8	35.2	35.3	34.9	None	Zhen Gong	Chengsheng Liao
16	17/5/2024	10:03 a.m	35	30	34.8	35.4	35.2	34.7	34.7	35.2	35.4	35	35.2	35.1	34.8	35	35.2	35.3	34.7	None	Zhen Gong	Chengsheng Liao
17	17/5/2024	3:27 p.m	35	30	35.3	35.3	35	34.9	34.7	34.7	34.7	35.4	34.7	35	35.2	35	35.4	35.1	34.8	None	Zhen Gong	Chengsheng Liao
18	17/5/2024	4:11 p.m	35	30	35.3	35	34.9	35.1	34.7	34.8	34.9	35.1	34.6	34.7	34.7	35.1	34.6	35.3	35.2	None	Zhen Gong	Chengsheng Liao
