# Supplementary material for: Design and implementation of corrosion-resistant multitasking cell stainers
Source: PLoS One. 2024 Oct 10;19(10):e0309334. doi: 10.1371/journal.pone.0309334 (PMC11466404; doi:10.1371/journal.pone.0309334)
Supplement: S3 Fig — (PDF) [file pone.0309334.s003.pdf]

Dyeing time data								
Numbered	Date	Time	Type of task	Number of frames	Dyeing time/min	Abnormal condition	Recorder	Reviewer
1	20/2/2023	9:00 a.m	Single-task	1	22.64	None	Leheng Li、Ming Mao	Chengsheng Liao
2	20/2/2023	9:31 a.m	Single-task	2	45.28	None	Leheng Li、Ming Mao	Chengsheng Liao
3	20/2/2023	10:23 a.m	Single-task	3	67.92	None	Leheng Li、Ming Mao	Chengsheng Liao
4	20/2/2023	3:13 p.m	Single-task	4	90.56	None	Leheng Li、Ming Mao	Chengsheng Liao
5	20/2/2023	4:57 p.m	Single-task	5	113.2	None	Leheng Li、Ming Mao	Chengsheng Liao
6	21/2/2023	8:57 a.m	Single-task	6	135.84	None	Leheng Li、Ming Mao	Chengsheng Liao
7	23/2/2023	3.05 p.m	Multitask	1	22.64	None	Leheng Li、Ming Mao	Chengsheng Liao
8	23/2/2023	3.34 p.m	Multitask	2	28.72	None	Leheng Li、Ming Mao	Chengsheng Liao
9	23/2/2023	4.15 p.m	Multitask	3	34.64	None	Leheng Li、Ming Mao	Chengsheng Liao
10	24/2/2023	9:09 a.m	Multitask	4	42.58	None	Leheng Li、Ming Mao	Chengsheng Liao
11	24/2/2023	10:00 a.m	Multitask	5	49.39	None	Leheng Li、Ming Mao	Chengsheng Liao
12	24/2/2023	3:16 p.m	Multitask	6	56.46	None	Leheng Li、Ming Mao	Chengsheng Liao
