# Supplementary material for: Design and implementation of corrosion-resistant multitasking cell stainers
Source: PLoS One. 2024 Oct 10;19(10):e0309334. doi: 10.1371/journal.pone.0309334 (PMC11466404; doi:10.1371/journal.pone.0309334)
Supplement: S4 Fig — (PDF) [file pone.0309334.s004.pdf]

Cell slide IOD scan data								
Numbered	Date	Time	Type of staining	Type of task	IOD	Abnormal condition	Recorder	Reviewer
1	18/5/2024	3:04 p.m	Pasteurization staining	Artificial	19.22	None	Zhen Gong	ChengSheng Liao
2	18/5/2024	3:04 p.m	Pasteurization staining	Artificial	18.83	None	Zhen Gong	ChengSheng Liao
3	18/5/2024	3:04 p.m	Pasteurization staining	Artificial	18.86	None	Zhen Gong	ChengSheng Liao
4	18/5/2024	3:04 p.m	Pasteurization staining	Artificial	19.3	None	Zhen Gong	ChengSheng Liao
5	18/5/2024	3:04 p.m	Pasteurization staining	Artificial	19.12	None	Zhen Gong	ChengSheng Liao
6	18/5/2024	3:04 p.m	Pasteurization staining	Artificial	19.29	None	Zhen Gong	ChengSheng Liao
7	18/5/2024	3:04 p.m	Pasteurization staining	Artificial	18.89	None	Zhen Gong	ChengSheng Liao
8	18/5/2024	3:04 p.m	Pasteurization staining	Artificial	18.7	None	Zhen Gong	ChengSheng Liao
9	18/5/2024	3:04 p.m	Pasteurization staining	Artificial	19.2	None	Zhen Gong	ChengSheng Liao
10	18/5/2024	3:04 p.m	Pasteurization staining	Artificial	19.31	None	Zhen Gong	ChengSheng Liao
11	18/5/2024	3:04 p.m	Pasteurization staining	Artificial	19.16	None	Zhen Gong	ChengSheng Liao
12	18/5/2024	3:04 p.m	Pasteurization staining	Artificial	19.09	None	Zhen Gong	ChengSheng Liao
13	18/5/2024	3:04 p.m	Pasteurization staining	Artificial	19.13	None	Zhen Gong	ChengSheng Liao
14	18/5/2024	3:04 p.m	Pasteurization staining	Artificial	19.37	None	Zhen Gong	ChengSheng Liao
15	18/5/2024	3:04 p.m	Pasteurization staining	Artificial	18.97	None	Zhen Gong	ChengSheng Liao
16	18/5/2024	3:04 p.m	Pasteurization staining	Artificial	19.39	None	Zhen Gong	ChengSheng Liao
17	18/5/2024	3:04 p.m	Pasteurization staining	Artificial	18.89	None	Zhen Gong	ChengSheng Liao
18	18/5/2024	3:04 p.m	Pasteurization staining	Artificial	19.07	None	Zhen Gong	ChengSheng Liao
19	18/5/2024	3:04 p.m	Pasteurization staining	Artificial	19.17	None	Zhen Gong	ChengSheng Liao
20	18/5/2024	3:04 p.m	Pasteurization staining	Artificial	18.67	None	Zhen Gong	ChengSheng Liao
21	18/5/2024	3:04 p.m	Pasteurization staining	Artificial	19.15	None	Zhen Gong	ChengSheng Liao
22	18/5/2024	3:04 p.m	Pasteurization staining	Artificial	18.83	None	Zhen Gong	ChengSheng Liao
23	18/5/2024	3:04 p.m	Pasteurization staining	Artificial	18.84	None	Zhen Gong	ChengSheng Liao
24	18/5/2024	3:04 p.m	Pasteurization staining	Artificial	18.93	None	Zhen Gong	ChengSheng Liao
25	18/5/2024	3:04 p.m	Pasteurization staining	Artificial	19.34	None	Zhen Gong	ChengSheng Liao
26	18/5/2024	3:04 p.m	Pasteurization staining	Artificial	18.84	None	Zhen Gong	ChengSheng Liao
27	18/5/2024	3:04 p.m	Pasteurization staining	Artificial	18.91	None	Zhen Gong	ChengSheng Liao
28	18/5/2024	3:04 p.m	Pasteurization staining	Artificial	18.67	None	Zhen Gong	ChengSheng Liao
29	18/5/2024	3:04 p.m	Pasteurization staining	Artificial	18.91	None	Zhen Gong	ChengSheng Liao
30	18/5/2024	3:04 p.m	Pasteurization staining	Artificial	18.66	None	Zhen Gong	ChengSheng Liao
31	18/5/2024	3:04 p.m	Pasteurization staining	Artificial	18.69	None	Zhen Gong	ChengSheng Liao
32	18/5/2024	3:04 p.m	Pasteurization staining	Artificial	19.15	None	Zhen Gong	ChengSheng Liao
33	18/5/2024	3:04 p.m	Pasteurization staining	Artificial	18.98	None	Zhen Gong	ChengSheng Liao
34	18/5/2024	3:04 p.m	Pasteurization staining	Artificial	18.97	None	Zhen Gong	ChengSheng Liao
35	18/5/2024	3:04 p.m	Pasteurization staining	Artificial	18.78	None	Zhen Gong	ChengSheng Liao
36	18/5/2024	3:04 p.m	Pasteurization staining	Artificial	18.82	None	Zhen Gong	ChengSheng Liao
37	18/5/2024	3:04 p.m	Pasteurization staining	Artificial	19.13	None	Zhen Gong	ChengSheng Liao
38	18/5/2024	3:04 p.m	Pasteurization staining	Artificial	18.89	None	Zhen Gong	ChengSheng Liao
39	18/5/2024	3:04 p.m	Pasteurization staining	Artificial	18.88	None	Zhen Gong	ChengSheng Liao
40	18/5/2024	3:04 p.m	Pasteurization staining	Artificial	19.34	None	Zhen Gong	ChengSheng Liao
41	18/5/2024	3:04 p.m	Pasteurization staining	Artificial	18.96	None	Zhen Gong	ChengSheng Liao
42	18/5/2024	3:04 p.m	Pasteurization staining	Artificial	19.43	None	Zhen Gong	ChengSheng Liao
43	18/5/2024	3:04 p.m	Pasteurization staining	Artificial	19.37	None	Zhen Gong	ChengSheng Liao
44	18/5/2024	3:04 p.m	Pasteurization staining	Artificial	18.88	None	Zhen Gong	ChengSheng Liao
45	18/5/2024	3:04 p.m	Pasteurization staining	Artificial	18.87	None	Zhen Gong	ChengSheng Liao
46	18/5/2024	3:04 p.m	Pasteurization staining	Artificial	18.99	None	Zhen Gong	ChengSheng Liao
47	18/5/2024	3:04 p.m	Pasteurization staining	Artificial	19.13	None	Zhen Gong	ChengSheng Liao
48	18/5/2024	3:04 p.m	Pasteurization staining	Artificial	19.44	None	Zhen Gong	ChengSheng Liao
49	18/5/2024	3:04 p.m	Pasteurization staining	Artificial	19.09	None	Zhen Gong	ChengSheng Liao
50	18/5/2024	3:04 p.m	Pasteurization staining	Artificial	19.07	None	Zhen Gong	ChengSheng Liao
51	19/5/2024	9:07 a.m	Pasteurization staining	Artificial	19.04	None	Zhen Gong	ChengSheng Liao
52	19/5/2024	9:07 a.m	Pasteurization staining	Artificial	18.86	None	Zhen Gong	ChengSheng Liao
53	19/5/2024	9:07 a.m	Pasteurization staining	Artificial	18.84	None	Zhen Gong	ChengSheng Liao
54	19/5/2024	9:07 a.m	Pasteurization staining	Artificial	18.72	None	Zhen Gong	ChengSheng Liao
55	19/5/2024	9:07 a.m	Pasteurization staining	Artificial	19.04	None	Zhen Gong	ChengSheng Liao
56	19/5/2024	9:07 a.m	Pasteurization staining	Artificial	19.07	None	Zhen Gong	ChengSheng Liao
57	19/5/2024	9:07 a.m	Pasteurization staining	Artificial	19.39	None	Zhen Gong	ChengSheng Liao
58	19/5/2024	9:07 a.m	Pasteurization staining	Artificial	19.36	None	Zhen Gong	ChengSheng Liao
59	19/5/2024	9:07 a.m	Pasteurization staining	Artificial	19.44	None	Zhen Gong	ChengSheng Liao
60	19/5/2024	9:07 a.m	Pasteurization staining	Artificial	19.35	None	Zhen Gong	ChengSheng Liao
61	19/5/2024	9:07 a.m	Pasteurization staining	Artificial	18.85	None	Zhen Gong	ChengSheng Liao
62	19/5/2024	9:07 a.m	Pasteurization staining	Artificial	18.84	None	Zhen Gong	ChengSheng Liao
63	19/5/2024	9:07 a.m	Pasteurization staining	Artificial	18.98	None	Zhen Gong	ChengSheng Liao
64	19/5/2024	9:07 a.m	Pasteurization staining	Artificial	18.79	None	Zhen Gong	ChengSheng Liao
65	19/5/2024	9:07 a.m	Pasteurization staining	Artificial	19.1	None	Zhen Gong	ChengSheng Liao
66	19/5/2024	9:07 a.m	Pasteurization staining	Artificial	18.97	None	Zhen Gong	ChengSheng Liao
67	19/5/2024	9:07 a.m	Pasteurization staining	Artificial	18.81	None	Zhen Gong	ChengSheng Liao

68	19/5/2024	9:07 a.m	Pasteurization staining	Artificial	18.96	None	Zhen Gong	ChengSheng Liao
69	19/5/2024	9:07 a.m	Pasteurization staining	Artificial	8.7	Dyeing failed	Zhen Gong	ChengSheng Liao
70	19/5/2024	9:07 a.m	Pasteurization staining	Artificial	18.91	None	Zhen Gong	ChengSheng Liao
71	19/5/2024	9:07 a.m	Pasteurization staining	Artificial	19.07	None	Zhen Gong	ChengSheng Liao
72	19/5/2024	9:07 a.m	Pasteurization staining	Artificial	19.37	None	Zhen Gong	ChengSheng Liao
73	19/5/2024	9:07 a.m	Pasteurization staining	Artificial	19.42	None	Zhen Gong	ChengSheng Liao
74	19/5/2024	9:07 a.m	Pasteurization staining	Artificial	18.8	None	Zhen Gong	ChengSheng Liao
75	19/5/2024	9:07 a.m	Pasteurization staining	Artificial	18.91	None	Zhen Gong	ChengSheng Liao
76	19/5/2024	9:07 a.m	Pasteurization staining	Artificial	18.9	None	Zhen Gong	ChengSheng Liao
77	19/5/2024	9:07 a.m	Pasteurization staining	Artificial	18.85	None	Zhen Gong	ChengSheng Liao
78	19/5/2024	9:07 a.m	Pasteurization staining	Artificial	18.66	None	Zhen Gong	ChengSheng Liao
79	19/5/2024	9:07 a.m	Pasteurization staining	Artificial	19.24	None	Zhen Gong	ChengSheng Liao
80	19/5/2024	9:07 a.m	Pasteurization staining	Artificial	19.44	None	Zhen Gong	ChengSheng Liao
81	19/5/2024	9:07 a.m	Pasteurization staining	Artificial	18.76	None	Zhen Gong	ChengSheng Liao
82	19/5/2024	9:07 a.m	Pasteurization staining	Artificial	18.86	None	Zhen Gong	ChengSheng Liao
83	19/5/2024	9:07 a.m	Pasteurization staining	Artificial	18.75	None	Zhen Gong	ChengSheng Liao
84	19/5/2024	9:07 a.m	Pasteurization staining	Artificial	19.13	None	Zhen Gong	ChengSheng Liao
85	19/5/2024	9:07 a.m	Pasteurization staining	Artificial	19.25	None	Zhen Gong	ChengSheng Liao
86	19/5/2024	9:07 a.m	Pasteurization staining	Artificial	18.91	None	Zhen Gong	ChengSheng Liao
87	19/5/2024	9:07 a.m	Pasteurization staining	Artificial	18.85	None	Zhen Gong	ChengSheng Liao
88	19/5/2024	9:07 a.m	Pasteurization staining	Artificial	19.36	None	Zhen Gong	ChengSheng Liao
89	19/5/2024	9:07 a.m	Pasteurization staining	Artificial	19.01	None	Zhen Gong	ChengSheng Liao
90	19/5/2024	9:07 a.m	Pasteurization staining	Artificial	18.85	None	Zhen Gong	ChengSheng Liao
91	19/5/2024	9:07 a.m	Pasteurization staining	Artificial	19.24	None	Zhen Gong	ChengSheng Liao
92	19/5/2024	9:07 a.m	Pasteurization staining	Artificial	19.35	None	Zhen Gong	ChengSheng Liao
93	19/5/2024	9:07 a.m	Pasteurization staining	Artificial	19.27	None	Zhen Gong	ChengSheng Liao
94	19/5/2024	9:07 a.m	Pasteurization staining	Artificial	19.38	None	Zhen Gong	ChengSheng Liao
95	19/5/2024	9:07 a.m	Pasteurization staining	Artificial	19.16	None	Zhen Gong	ChengSheng Liao
96	19/5/2024	9:07 a.m	Pasteurization staining	Artificial	19.09	None	Zhen Gong	ChengSheng Liao
97	19/5/2024	9:07 a.m	Pasteurization staining	Artificial	19.03	None	Zhen Gong	ChengSheng Liao
98	19/5/2024	9:07 a.m	Pasteurization staining	Artificial	19.23	None	Zhen Gong	ChengSheng Liao
99	19/5/2024	9:07 a.m	Pasteurization staining	Artificial	18.86	None	Zhen Gong	ChengSheng Liao
100	19/5/2024	9:07 a.m	Pasteurization staining	Artificial	19.04	None	Zhen Gong	ChengSheng Liao

Cell slide IOD scan data								
Numbered	Date	Time	Type of staining	Type of task	IOD	Abnormal condition	Recorder	Reviewer
1	19/5/2024	3:16 p.m	Pasteurization staining	Multitask	19.55	None	Zhen Gong	ChengSheng Liao
2	19/5/2024	3:16 p.m	Pasteurization staining	Multitask	19.49	None	Zhen Gong	ChengSheng Liao
3	19/5/2024	3:16 p.m	Pasteurization staining	Multitask	19.29	None	Zhen Gong	ChengSheng Liao
4	19/5/2024	3:16 p.m	Pasteurization staining	Multitask	19.32	None	Zhen Gong	ChengSheng Liao
5	19/5/2024	3:16 p.m	Pasteurization staining	Multitask	19.39	None	Zhen Gong	ChengSheng Liao
6	19/5/2024	3:16 p.m	Pasteurization staining	Multitask	19.29	None	Zhen Gong	ChengSheng Liao
7	19/5/2024	3:16 p.m	Pasteurization staining	Multitask	19.28	None	Zhen Gong	ChengSheng Liao
8	19/5/2024	3:16 p.m	Pasteurization staining	Multitask	19.28	None	Zhen Gong	ChengSheng Liao
9	19/5/2024	3:16 p.m	Pasteurization staining	Multitask	19.6	None	Zhen Gong	ChengSheng Liao
10	19/5/2024	3:16 p.m	Pasteurization staining	Multitask	19.67	None	Zhen Gong	ChengSheng Liao
11	19/5/2024	3:16 p.m	Pasteurization staining	Multitask	19.7	None	Zhen Gong	ChengSheng Liao
12	19/5/2024	3:16 p.m	Pasteurization staining	Multitask	19.24	None	Zhen Gong	ChengSheng Liao
13	19/5/2024	3:16 p.m	Pasteurization staining	Multitask	19.5	None	Zhen Gong	ChengSheng Liao
14	19/5/2024	3:16 p.m	Pasteurization staining	Multitask	19.29	None	Zhen Gong	ChengSheng Liao
15	19/5/2024	3:16 p.m	Pasteurization staining	Multitask	19.21	None	Zhen Gong	ChengSheng Liao
16	19/5/2024	3:16 p.m	Pasteurization staining	Multitask	19.38	None	Zhen Gong	ChengSheng Liao
17	19/5/2024	3:16 p.m	Pasteurization staining	Multitask	19.42	None	Zhen Gong	ChengSheng Liao
18	19/5/2024	3:16 p.m	Pasteurization staining	Multitask	19.37	None	Zhen Gong	ChengSheng Liao
19	19/5/2024	3:16 p.m	Pasteurization staining	Multitask	19.12	None	Zhen Gong	ChengSheng Liao
20	19/5/2024	3:16 p.m	Pasteurization staining	Multitask	19.63	None	Zhen Gong	ChengSheng Liao
21	19/5/2024	3:16 p.m	Pasteurization staining	Multitask	19.36	None	Zhen Gong	ChengSheng Liao
22	19/5/2024	3:16 p.m	Pasteurization staining	Multitask	19.27	None	Zhen Gong	ChengSheng Liao
23	19/5/2024	3:16 p.m	Pasteurization staining	Multitask	19.29	None	Zhen Gong	ChengSheng Liao
24	19/5/2024	3:16 p.m	Pasteurization staining	Multitask	19.27	None	Zhen Gong	ChengSheng Liao
25	19/5/2024	3:16 p.m	Pasteurization staining	Multitask	19.51	None	Zhen Gong	ChengSheng Liao
26	19/5/2024	3:16 p.m	Pasteurization staining	Multitask	19.53	None	Zhen Gong	ChengSheng Liao
27	19/5/2024	3:16 p.m	Pasteurization staining	Multitask	19.59	None	Zhen Gong	ChengSheng Liao
28	19/5/2024	3:16 p.m	Pasteurization staining	Multitask	19.19	None	Zhen Gong	ChengSheng Liao
29	19/5/2024	3:16 p.m	Pasteurization staining	Multitask	19.16	None	Zhen Gong	ChengSheng Liao
30	19/5/2024	3:16 p.m	Pasteurization staining	Multitask	19.46	None	Zhen Gong	ChengSheng Liao
31	19/5/2024	3:16 p.m	Pasteurization staining	Multitask	19.35	None	Zhen Gong	ChengSheng Liao
32	19/5/2024	3:16 p.m	Pasteurization staining	Multitask	19.55	None	Zhen Gong	ChengSheng Liao
33	19/5/2024	3:16 p.m	Pasteurization staining	Multitask	19.25	None	Zhen Gong	ChengSheng Liao
34	19/5/2024	3:16 p.m	Pasteurization staining	Multitask	19.47	None	Zhen Gong	ChengSheng Liao
35	19/5/2024	3:16 p.m	Pasteurization staining	Multitask	19.33	None	Zhen Gong	ChengSheng Liao
36	19/5/2024	3:16 p.m	Pasteurization staining	Multitask	19.42	None	Zhen Gong	ChengSheng Liao
37	19/5/2024	3:16 p.m	Pasteurization staining	Multitask	19.69	None	Zhen Gong	ChengSheng Liao
38	19/5/2024	3:16 p.m	Pasteurization staining	Multitask	19.7	None	Zhen Gong	ChengSheng Liao
39	19/5/2024	3:16 p.m	Pasteurization staining	Multitask	19.47	None	Zhen Gong	ChengSheng Liao
40	19/5/2024	3:16 p.m	Pasteurization staining	Multitask	19.52	None	Zhen Gong	ChengSheng Liao
41	19/5/2024	3:16 p.m	Pasteurization staining	Multitask	19.67	None	Zhen Gong	ChengSheng Liao
42	19/5/2024	3:16 p.m	Pasteurization staining	Multitask	19.54	None	Zhen Gong	ChengSheng Liao
43	19/5/2024	3:16 p.m	Pasteurization staining	Multitask	19.36	None	Zhen Gong	ChengSheng Liao
44	19/5/2024	3:16 p.m	Pasteurization staining	Multitask	19.6	None	Zhen Gong	ChengSheng Liao
45	19/5/2024	3:16 p.m	Pasteurization staining	Multitask	19.53	None	Zhen Gong	ChengSheng Liao
46	19/5/2024	3:16 p.m	Pasteurization staining	Multitask	19.5	None	Zhen Gong	ChengSheng Liao
47	19/5/2024	3:16 p.m	Pasteurization staining	Multitask	19.41	None	Zhen Gong	ChengSheng Liao
48	19/5/2024	3:16 p.m	Pasteurization staining	Multitask	19.31	None	Zhen Gong	ChengSheng Liao
49	19/5/2024	3:16 p.m	Pasteurization staining	Multitask	19.17	None	Zhen Gong	ChengSheng Liao
50	19/5/2024	3:16 p.m	Pasteurization staining	Multitask	19.65	None	Zhen Gong	ChengSheng Liao
51	20/5/2024	9:06 a.m	Pasteurization staining	Multitask	19.24	None	Zhen Gong	ChengSheng Liao
52	20/5/2024	9:06 a.m	Pasteurization staining	Multitask	19.26	None	Zhen Gong	ChengSheng Liao
53	20/5/2024	9:06 a.m	Pasteurization staining	Multitask	19.34	None	Zhen Gong	ChengSheng Liao
54	20/5/2024	9:06 a.m	Pasteurization staining	Multitask	19.65	None	Zhen Gong	ChengSheng Liao
55	20/5/2024	9:06 a.m	Pasteurization staining	Multitask	19.13	None	Zhen Gong	ChengSheng Liao
56	20/5/2024	9:06 a.m	Pasteurization staining	Multitask	19.23	None	Zhen Gong	ChengSheng Liao
57	20/5/2024	9:06 a.m	Pasteurization staining	Multitask	19.68	None	Zhen Gong	ChengSheng Liao
58	20/5/2024	9:06 a.m	Pasteurization staining	Multitask	19.29	None	Zhen Gong	ChengSheng Liao
59	20/5/2024	9:06 a.m	Pasteurization staining	Multitask	19.15	None	Zhen Gong	ChengSheng Liao
60	20/5/2024	9:06 a.m	Pasteurization staining	Multitask	19.57	None	Zhen Gong	ChengSheng Liao
61	20/5/2024	9:06 a.m	Pasteurization staining	Multitask	19.24	None	Zhen Gong	ChengSheng Liao
62	20/5/2024	9:06 a.m	Pasteurization staining	Multitask	19.59	None	Zhen Gong	ChengSheng Liao
63	20/5/2024	9:06 a.m	Pasteurization staining	Multitask	19.59	None	Zhen Gong	ChengSheng Liao
64	20/5/2024	9:06 a.m	Pasteurization staining	Multitask	19.25	None	Zhen Gong	ChengSheng Liao
65	20/5/2024	9:06 a.m	Pasteurization staining	Multitask	19.28	None	Zhen Gong	ChengSheng Liao
66	20/5/2024	9:06 a.m	Pasteurization staining	Multitask	19.59	None	Zhen Gong	ChengSheng Liao
67	20/5/2024	9:06 a.m	Pasteurization staining	Multitask	19.29	None	Zhen Gong	ChengSheng Liao

68	20/5/2024	9:06 a.m	Pasteurization staining	Multitask	19.6	None	Zhen Gong	ChengSheng Liao
69	20/5/2024	9:06 a.m	Pasteurization staining	Multitask	19.37	None	Zhen Gong	ChengSheng Liao
70	20/5/2024	9:06 a.m	Pasteurization staining	Multitask	19.16	None	Zhen Gong	ChengSheng Liao
71	20/5/2024	9:06 a.m	Pasteurization staining	Multitask	19.66	None	Zhen Gong	ChengSheng Liao
72	20/5/2024	9:06 a.m	Pasteurization staining	Multitask	19.61	None	Zhen Gong	ChengSheng Liao
73	20/5/2024	9:06 a.m	Pasteurization staining	Multitask	19.32	None	Zhen Gong	ChengSheng Liao
74	20/5/2024	9:06 a.m	Pasteurization staining	Multitask	19.28	None	Zhen Gong	ChengSheng Liao
75	20/5/2024	9:06 a.m	Pasteurization staining	Multitask	19.53	None	Zhen Gong	ChengSheng Liao
76	20/5/2024	9:06 a.m	Pasteurization staining	Multitask	19.35	None	Zhen Gong	ChengSheng Liao
77	20/5/2024	9:06 a.m	Pasteurization staining	Multitask	19.36	None	Zhen Gong	ChengSheng Liao
78	20/5/2024	9:06 a.m	Pasteurization staining	Multitask	19.37	None	Zhen Gong	ChengSheng Liao
79	20/5/2024	9:06 a.m	Pasteurization staining	Multitask	19.23	None	Zhen Gong	ChengSheng Liao
80	20/5/2024	9:06 a.m	Pasteurization staining	Multitask	19.63	None	Zhen Gong	ChengSheng Liao
81	20/5/2024	9:06 a.m	Pasteurization staining	Multitask	19.31	None	Zhen Gong	ChengSheng Liao
82	20/5/2024	9:06 a.m	Pasteurization staining	Multitask	19.44	None	Zhen Gong	ChengSheng Liao
83	20/5/2024	9:06 a.m	Pasteurization staining	Multitask	19.27	None	Zhen Gong	ChengSheng Liao
84	20/5/2024	9:06 a.m	Pasteurization staining	Multitask	19.59	None	Zhen Gong	ChengSheng Liao
85	20/5/2024	9:06 a.m	Pasteurization staining	Multitask	19.59	None	Zhen Gong	ChengSheng Liao
86	20/5/2024	9:06 a.m	Pasteurization staining	Multitask	19.36	None	Zhen Gong	ChengSheng Liao
87	20/5/2024	9:06 a.m	Pasteurization staining	Multitask	19.45	None	Zhen Gong	ChengSheng Liao
88	20/5/2024	9:06 a.m	Pasteurization staining	Multitask	19.33	None	Zhen Gong	ChengSheng Liao
89	20/5/2024	9:06 a.m	Pasteurization staining	Multitask	19.26	None	Zhen Gong	ChengSheng Liao
90	20/5/2024	9:06 a.m	Pasteurization staining	Multitask	19.28	None	Zhen Gong	ChengSheng Liao
91	20/5/2024	9:06 a.m	Pasteurization staining	Multitask	19.41	None	Zhen Gong	ChengSheng Liao
92	20/5/2024	9:06 a.m	Pasteurization staining	Multitask	19.34	None	Zhen Gong	ChengSheng Liao
93	20/5/2024	9:06 a.m	Pasteurization staining	Multitask	19.65	None	Zhen Gong	ChengSheng Liao
94	20/5/2024	9:06 a.m	Pasteurization staining	Multitask	19.59	None	Zhen Gong	ChengSheng Liao
95	20/5/2024	9:06 a.m	Pasteurization staining	Multitask	19.53	None	Zhen Gong	ChengSheng Liao
96	20/5/2024	9:06 a.m	Pasteurization staining	Multitask	19.61	None	Zhen Gong	ChengSheng Liao
97	20/5/2024	9:06 a.m	Pasteurization staining	Multitask	19.66	None	Zhen Gong	ChengSheng Liao
98	20/5/2024	9:06 a.m	Pasteurization staining	Multitask	19.44	None	Zhen Gong	ChengSheng Liao
99	20/5/2024	9:06 a.m	Pasteurization staining	Multitask	19.35	None	Zhen Gong	ChengSheng Liao
100	20/5/2024	9:06 a.m	Pasteurization staining	Multitask	19.56	None	Zhen Gong	ChengSheng Liao

Cell slide IOD scan data								
Numbered	Date	Time	Type of staining	Type of task	IOD	Abnormal condition	Recorder	Reviewer
1	20/5/2024	3:01 p.m	Pasteurization staining	Single-task	18.59	None	Zhen Gong	ChengSheng Liao
2	20/5/2024	3:01 p.m	Pasteurization staining	Single-task	18.43	None	Zhen Gong	ChengSheng Liao
3	20/5/2024	3:01 p.m	Pasteurization staining	Single-task	18.38	None	Zhen Gong	ChengSheng Liao
4	20/5/2024	3:01 p.m	Pasteurization staining	Single-task	18.65	None	Zhen Gong	ChengSheng Liao
5	20/5/2024	3:01 p.m	Pasteurization staining	Single-task	18.71	None	Zhen Gong	ChengSheng Liao
6	20/5/2024	3:01 p.m	Pasteurization staining	Single-task	18.61	None	Zhen Gong	ChengSheng Liao
7	20/5/2024	3:01 p.m	Pasteurization staining	Single-task	18.52	None	Zhen Gong	ChengSheng Liao
8	20/5/2024	3:01 p.m	Pasteurization staining	Single-task	18.58	None	Zhen Gong	ChengSheng Liao
9	20/5/2024	3:01 p.m	Pasteurization staining	Single-task	18.73	None	Zhen Gong	ChengSheng Liao
10	20/5/2024	3:01 p.m	Pasteurization staining	Single-task	18.86	None	Zhen Gong	ChengSheng Liao
11	20/5/2024	3:01 p.m	Pasteurization staining	Single-task	18.67	None	Zhen Gong	ChengSheng Liao
12	20/5/2024	3:01 p.m	Pasteurization staining	Single-task	18.48	None	Zhen Gong	ChengSheng Liao
13	20/5/2024	3:01 p.m	Pasteurization staining	Single-task	18.85	None	Zhen Gong	ChengSheng Liao
14	20/5/2024	3:01 p.m	Pasteurization staining	Single-task	18.59	None	Zhen Gong	ChengSheng Liao
15	20/5/2024	3:01 p.m	Pasteurization staining	Single-task	18.39	None	Zhen Gong	ChengSheng Liao
16	20/5/2024	3:01 p.m	Pasteurization staining	Single-task	18.72	None	Zhen Gong	ChengSheng Liao
17	20/5/2024	3:01 p.m	Pasteurization staining	Single-task	18.76	None	Zhen Gong	ChengSheng Liao
18	20/5/2024	3:01 p.m	Pasteurization staining	Single-task	18.71	None	Zhen Gong	ChengSheng Liao
19	20/5/2024	3:01 p.m	Pasteurization staining	Single-task	18.39	None	Zhen Gong	ChengSheng Liao
20	20/5/2024	3:01 p.m	Pasteurization staining	Single-task	18.49	None	Zhen Gong	ChengSheng Liao
21	20/5/2024	3:01 p.m	Pasteurization staining	Single-task	18.84	None	Zhen Gong	ChengSheng Liao
22	20/5/2024	3:01 p.m	Pasteurization staining	Single-task	18.61	None	Zhen Gong	ChengSheng Liao
23	20/5/2024	3:01 p.m	Pasteurization staining	Single-task	18.76	None	Zhen Gong	ChengSheng Liao
24	20/5/2024	3:01 p.m	Pasteurization staining	Single-task	18.67	None	Zhen Gong	ChengSheng Liao
25	20/5/2024	3:01 p.m	Pasteurization staining	Single-task	18.87	None	Zhen Gong	ChengSheng Liao
26	20/5/2024	3:01 p.m	Pasteurization staining	Single-task	18.78	None	Zhen Gong	ChengSheng Liao
27	20/5/2024	3:01 p.m	Pasteurization staining	Single-task	18.83	None	Zhen Gong	ChengSheng Liao
28	20/5/2024	3:01 p.m	Pasteurization staining	Single-task	18.58	None	Zhen Gong	ChengSheng Liao
29	20/5/2024	3:01 p.m	Pasteurization staining	Single-task	18.86	None	Zhen Gong	ChengSheng Liao
30	20/5/2024	3:01 p.m	Pasteurization staining	Single-task	18.48	None	Zhen Gong	ChengSheng Liao
31	20/5/2024	3:01 p.m	Pasteurization staining	Single-task	18.61	None	Zhen Gong	ChengSheng Liao
32	20/5/2024	3:01 p.m	Pasteurization staining	Single-task	18.58	None	Zhen Gong	ChengSheng Liao
33	20/5/2024	3:01 p.m	Pasteurization staining	Single-task	18.44	None	Zhen Gong	ChengSheng Liao
34	20/5/2024	3:01 p.m	Pasteurization staining	Single-task	18.36	None	Zhen Gong	ChengSheng Liao
35	20/5/2024	3:01 p.m	Pasteurization staining	Single-task	18.57	None	Zhen Gong	ChengSheng Liao
36	20/5/2024	3:01 p.m	Pasteurization staining	Single-task	18.38	None	Zhen Gong	ChengSheng Liao
37	20/5/2024	3:01 p.m	Pasteurization staining	Single-task	18.38	None	Zhen Gong	ChengSheng Liao
38	20/5/2024	3:01 p.m	Pasteurization staining	Single-task	18.66	None	Zhen Gong	ChengSheng Liao
39	20/5/2024	3:01 p.m	Pasteurization staining	Single-task	18.73	None	Zhen Gong	ChengSheng Liao
40	20/5/2024	3:01 p.m	Pasteurization staining	Single-task	18.35	None	Zhen Gong	ChengSheng Liao
41	20/5/2024	3:01 p.m	Pasteurization staining	Single-task	18.61	None	Zhen Gong	ChengSheng Liao
42	20/5/2024	3:01 p.m	Pasteurization staining	Single-task	18.52	None	Zhen Gong	ChengSheng Liao
43	20/5/2024	3:01 p.m	Pasteurization staining	Single-task	18.46	None	Zhen Gong	ChengSheng Liao
44	20/5/2024	3:01 p.m	Pasteurization staining	Single-task	18.46	None	Zhen Gong	ChengSheng Liao
45	20/5/2024	3:01 p.m	Pasteurization staining	Single-task	18.72	None	Zhen Gong	ChengSheng Liao
46	20/5/2024	3:01 p.m	Pasteurization staining	Single-task	18.89	None	Zhen Gong	ChengSheng Liao
47	20/5/2024	3:01 p.m	Pasteurization staining	Single-task	18.76	None	Zhen Gong	ChengSheng Liao
48	20/5/2024	3:01 p.m	Pasteurization staining	Single-task	18.56	None	Zhen Gong	ChengSheng Liao
49	20/5/2024	3:01 p.m	Pasteurization staining	Single-task	18.65	None	Zhen Gong	ChengSheng Liao
50	20/5/2024	3:01 p.m	Pasteurization staining	Single-task	18.54	None	Zhen Gong	ChengSheng Liao
51	21/5/2024	9:04 a.m	Pasteurization staining	Single-task	18.66	None	Zhen Gong	ChengSheng Liao
52	21/5/2024	9:04 a.m	Pasteurization staining	Single-task	18.56	None	Zhen Gong	ChengSheng Liao
53	21/5/2024	9:04 a.m	Pasteurization staining	Single-task	18.49	None	Zhen Gong	ChengSheng Liao
54	21/5/2024	9:04 a.m	Pasteurization staining	Single-task	18.69	None	Zhen Gong	ChengSheng Liao
55	21/5/2024	9:04 a.m	Pasteurization staining	Single-task	18.54	None	Zhen Gong	ChengSheng Liao
56	21/5/2024	9:04 a.m	Pasteurization staining	Single-task	18.68	None	Zhen Gong	ChengSheng Liao
57	21/5/2024	9:04 a.m	Pasteurization staining	Single-task	18.31	None	Zhen Gong	ChengSheng Liao
58	21/5/2024	9:04 a.m	Pasteurization staining	Single-task	18.39	None	Zhen Gong	ChengSheng Liao
59	21/5/2024	9:04 a.m	Pasteurization staining	Single-task	18.43	None	Zhen Gong	ChengSheng Liao
60	21/5/2024	9:04 a.m	Pasteurization staining	Single-task	18.71	None	Zhen Gong	ChengSheng Liao
61	21/5/2024	9:04 a.m	Pasteurization staining	Single-task	18.78	None	Zhen Gong	ChengSheng Liao
62	21/5/2024	9:04 a.m	Pasteurization staining	Single-task	18.34	None	Zhen Gong	ChengSheng Liao
63	21/5/2024	9:04 a.m	Pasteurization staining	Single-task	18.45	None	Zhen Gong	ChengSheng Liao
64	21/5/2024	9:04 a.m	Pasteurization staining	Single-task	18.4	None	Zhen Gong	ChengSheng Liao
65	21/5/2024	9:04 a.m	Pasteurization staining	Single-task	18.35	None	Zhen Gong	ChengSheng Liao
66	21/5/2024	9:04 a.m	Pasteurization staining	Single-task	18.68	None	Zhen Gong	ChengSheng Liao
67	21/5/2024	9:04 a.m	Pasteurization staining	Single-task	18.58	None	Zhen Gong	ChengSheng Liao

68	21/5/2024	9:04 a.m	Pasteurization staining	Single-task	18.69	None	Zhen Gong	ChengSheng Liao
69	21/5/2024	9:04 a.m	Pasteurization staining	Single-task	18.72	None	Zhen Gong	ChengSheng Liao
70	21/5/2024	9:04 a.m	Pasteurization staining	Single-task	18.66	None	Zhen Gong	ChengSheng Liao
71	21/5/2024	9:04 a.m	Pasteurization staining	Single-task	18.62	None	Zhen Gong	ChengSheng Liao
72	21/5/2024	9:04 a.m	Pasteurization staining	Single-task	18.79	None	Zhen Gong	ChengSheng Liao
73	21/5/2024	9:04 a.m	Pasteurization staining	Single-task	18.47	None	Zhen Gong	ChengSheng Liao
74	21/5/2024	9:04 a.m	Pasteurization staining	Single-task	18.32	None	Zhen Gong	ChengSheng Liao
75	21/5/2024	9:04 a.m	Pasteurization staining	Single-task	18.58	None	Zhen Gong	ChengSheng Liao
76	21/5/2024	9:04 a.m	Pasteurization staining	Single-task	18.82	None	Zhen Gong	ChengSheng Liao
77	21/5/2024	9:04 a.m	Pasteurization staining	Single-task	18.73	None	Zhen Gong	ChengSheng Liao
78	21/5/2024	9:04 a.m	Pasteurization staining	Single-task	18.57	None	Zhen Gong	ChengSheng Liao
79	21/5/2024	9:04 a.m	Pasteurization staining	Single-task	18.56	None	Zhen Gong	ChengSheng Liao
80	21/5/2024	9:04 a.m	Pasteurization staining	Single-task	18.83	None	Zhen Gong	ChengSheng Liao
81	21/5/2024	9:04 a.m	Pasteurization staining	Single-task	18.58	None	Zhen Gong	ChengSheng Liao
82	21/5/2024	9:04 a.m	Pasteurization staining	Single-task	18.51	None	Zhen Gong	ChengSheng Liao
83	21/5/2024	9:04 a.m	Pasteurization staining	Single-task	18.68	None	Zhen Gong	ChengSheng Liao
84	21/5/2024	9:04 a.m	Pasteurization staining	Single-task	18.51	None	Zhen Gong	ChengSheng Liao
85	21/5/2024	9:04 a.m	Pasteurization staining	Single-task	18.85	None	Zhen Gong	ChengSheng Liao
86	21/5/2024	9:04 a.m	Pasteurization staining	Single-task	18.63	None	Zhen Gong	ChengSheng Liao
87	21/5/2024	9:04 a.m	Pasteurization staining	Single-task	18.38	None	Zhen Gong	ChengSheng Liao
88	21/5/2024	9:04 a.m	Pasteurization staining	Single-task	18.59	None	Zhen Gong	ChengSheng Liao
89	21/5/2024	9:04 a.m	Pasteurization staining	Single-task	18.58	None	Zhen Gong	ChengSheng Liao
90	21/5/2024	9:04 a.m	Pasteurization staining	Single-task	18.62	None	Zhen Gong	ChengSheng Liao
91	21/5/2024	9:04 a.m	Pasteurization staining	Single-task	18.52	None	Zhen Gong	ChengSheng Liao
92	21/5/2024	9:04 a.m	Pasteurization staining	Single-task	18.43	None	Zhen Gong	ChengSheng Liao
93	21/5/2024	9:04 a.m	Pasteurization staining	Single-task	18.61	None	Zhen Gong	ChengSheng Liao
94	21/5/2024	9:04 a.m	Pasteurization staining	Single-task	18.63	None	Zhen Gong	ChengSheng Liao
95	21/5/2024	9:04 a.m	Pasteurization staining	Single-task	18.41	None	Zhen Gong	ChengSheng Liao
96	21/5/2024	9:04 a.m	Pasteurization staining	Single-task	18.32	None	Zhen Gong	ChengSheng Liao
97	21/5/2024	9:04 a.m	Pasteurization staining	Single-task	18.62	None	Zhen Gong	ChengSheng Liao
98	21/5/2024	9:04 a.m	Pasteurization staining	Single-task	18.64	None	Zhen Gong	ChengSheng Liao
99	21/5/2024	9:04 a.m	Pasteurization staining	Single-task	18.58	None	Zhen Gong	ChengSheng Liao
100	21/5/2024	9:04 a.m	Pasteurization staining	Single-task	18.68	None	Zhen Gong	ChengSheng Liao

Cell slide IOD scan data								
Numbered	Date	Time	Type of staining	Type of task	IOD	Abnormal condition	Recorder	Reviewer
1	21/5/2024	3:03 p.m	New composite staining	Artificial	19.09	None	Zhen Gong	ChengSheng Liao
2	21/5/2024	3:03 p.m	New composite staining	Artificial	18.93	None	Zhen Gong	ChengSheng Liao
3	21/5/2024	3:03 p.m	New composite staining	Artificial	19.29	None	Zhen Gong	ChengSheng Liao
4	21/5/2024	3:03 p.m	New composite staining	Artificial	18.9	None	Zhen Gong	ChengSheng Liao
5	21/5/2024	3:03 p.m	New composite staining	Artificial	19.22	None	Zhen Gong	ChengSheng Liao
6	21/5/2024	3:03 p.m	New composite staining	Artificial	19.13	None	Zhen Gong	ChengSheng Liao
7	21/5/2024	3:03 p.m	New composite staining	Artificial	19.03	None	Zhen Gong	ChengSheng Liao
8	21/5/2024	3:03 p.m	New composite staining	Artificial	18.76	None	Zhen Gong	ChengSheng Liao
9	21/5/2024	3:03 p.m	New composite staining	Artificial	19.25	None	Zhen Gong	ChengSheng Liao
10	21/5/2024	3:03 p.m	New composite staining	Artificial	19.3	None	Zhen Gong	ChengSheng Liao
11	21/5/2024	3:03 p.m	New composite staining	Artificial	19.27	None	Zhen Gong	ChengSheng Liao
12	21/5/2024	3:03 p.m	New composite staining	Artificial	18.79	None	Zhen Gong	ChengSheng Liao
13	21/5/2024	3:03 p.m	New composite staining	Artificial	18.71	None	Zhen Gong	ChengSheng Liao
14	21/5/2024	3:03 p.m	New composite staining	Artificial	19.03	None	Zhen Gong	ChengSheng Liao
15	21/5/2024	3:03 p.m	New composite staining	Artificial	19.34	None	Zhen Gong	ChengSheng Liao
16	21/5/2024	3:03 p.m	New composite staining	Artificial	18.71	None	Zhen Gong	ChengSheng Liao
17	21/5/2024	3:03 p.m	New composite staining	Artificial	18.72	None	Zhen Gong	ChengSheng Liao
18	21/5/2024	3:03 p.m	New composite staining	Artificial	19.04	None	Zhen Gong	ChengSheng Liao
19	21/5/2024	3:03 p.m	New composite staining	Artificial	18.7	None	Zhen Gong	ChengSheng Liao
20	21/5/2024	3:03 p.m	New composite staining	Artificial	19.12	None	Zhen Gong	ChengSheng Liao
21	21/5/2024	3:03 p.m	New composite staining	Artificial	18.73	None	Zhen Gong	ChengSheng Liao
22	21/5/2024	3:03 p.m	New composite staining	Artificial	19.19	None	Zhen Gong	ChengSheng Liao
23	21/5/2024	3:03 p.m	New composite staining	Artificial	19.01	None	Zhen Gong	ChengSheng Liao
24	21/5/2024	3:03 p.m	New composite staining	Artificial	18.98	None	Zhen Gong	ChengSheng Liao
25	21/5/2024	3:03 p.m	New composite staining	Artificial	18.91	None	Zhen Gong	ChengSheng Liao
26	21/5/2024	3:03 p.m	New composite staining	Artificial	18.76	None	Zhen Gong	ChengSheng Liao
27	21/5/2024	3:03 p.m	New composite staining	Artificial	19.17	None	Zhen Gong	ChengSheng Liao
28	21/5/2024	3:03 p.m	New composite staining	Artificial	19	None	Zhen Gong	ChengSheng Liao
29	21/5/2024	3:03 p.m	New composite staining	Artificial	18.67	None	Zhen Gong	ChengSheng Liao
30	21/5/2024	3:03 p.m	New composite staining	Artificial	18.93	None	Zhen Gong	ChengSheng Liao
31	21/5/2024	3:03 p.m	New composite staining	Artificial	19.1	None	Zhen Gong	ChengSheng Liao
32	21/5/2024	3:03 p.m	New composite staining	Artificial	18.86	None	Zhen Gong	ChengSheng Liao
33	21/5/2024	3:03 p.m	New composite staining	Artificial	19.15	None	Zhen Gong	ChengSheng Liao
34	21/5/2024	3:03 p.m	New composite staining	Artificial	18.98	None	Zhen Gong	ChengSheng Liao
35	21/5/2024	3:03 p.m	New composite staining	Artificial	8.98	Dyeing failed	Zhen Gong	ChengSheng Liao
36	21/5/2024	3:03 p.m	New composite staining	Artificial	18.7	None	Zhen Gong	ChengSheng Liao
37	21/5/2024	3:03 p.m	New composite staining	Artificial	19.29	None	Zhen Gong	ChengSheng Liao
38	21/5/2024	3:03 p.m	New composite staining	Artificial	18.91	None	Zhen Gong	ChengSheng Liao
39	21/5/2024	3:03 p.m	New composite staining	Artificial	18.68	None	Zhen Gong	ChengSheng Liao
40	21/5/2024	3:03 p.m	New composite staining	Artificial	18.96	None	Zhen Gong	ChengSheng Liao
41	21/5/2024	3:03 p.m	New composite staining	Artificial	19.16	None	Zhen Gong	ChengSheng Liao
42	21/5/2024	3:03 p.m	New composite staining	Artificial	18.97	None	Zhen Gong	ChengSheng Liao
43	21/5/2024	3:03 p.m	New composite staining	Artificial	19.18	None	Zhen Gong	ChengSheng Liao
44	21/5/2024	3:03 p.m	New composite staining	Artificial	18.74	None	Zhen Gong	ChengSheng Liao
45	21/5/2024	3:03 p.m	New composite staining	Artificial	19.15	None	Zhen Gong	ChengSheng Liao
46	21/5/2024	3:03 p.m	New composite staining	Artificial	18.82	None	Zhen Gong	ChengSheng Liao
47	21/5/2024	3:03 p.m	New composite staining	Artificial	18.73	None	Zhen Gong	ChengSheng Liao
48	21/5/2024	3:03 p.m	New composite staining	Artificial	18.87	None	Zhen Gong	ChengSheng Liao
49	21/5/2024	3:03 p.m	New composite staining	Artificial	19.08	None	Zhen Gong	ChengSheng Liao
50	21/5/2024	3:03 p.m	New composite staining	Artificial	19.23	None	Zhen Gong	ChengSheng Liao
51	22/5/2024	9:02 a.m	New composite staining	Artificial	18.72	None	Zhen Gong	ChengSheng Liao
52	22/5/2024	9:02 a.m	New composite staining	Artificial	19.25	None	Zhen Gong	ChengSheng Liao
53	22/5/2024	9:02 a.m	New composite staining	Artificial	19.02	None	Zhen Gong	ChengSheng Liao
54	22/5/2024	9:02 a.m	New composite staining	Artificial	19.04	None	Zhen Gong	ChengSheng Liao
55	22/5/2024	9:02 a.m	New composite staining	Artificial	18.72	None	Zhen Gong	ChengSheng Liao
56	22/5/2024	9:02 a.m	New composite staining	Artificial	18.71	None	Zhen Gong	ChengSheng Liao
57	22/5/2024	9:02 a.m	New composite staining	Artificial	19.13	None	Zhen Gong	ChengSheng Liao
58	22/5/2024	9:02 a.m	New composite staining	Artificial	18.99	None	Zhen Gong	ChengSheng Liao
59	22/5/2024	9:02 a.m	New composite staining	Artificial	19.33	None	Zhen Gong	ChengSheng Liao
60	22/5/2024	9:02 a.m	New composite staining	Artificial	19.25	None	Zhen Gong	ChengSheng Liao
61	22/5/2024	9:02 a.m	New composite staining	Artificial	18.98	None	Zhen Gong	ChengSheng Liao
62	22/5/2024	9:02 a.m	New composite staining	Artificial	18.9	None	Zhen Gong	ChengSheng Liao
63	22/5/2024	9:02 a.m	New composite staining	Artificial	7.39	Dyeing failed	Zhen Gong	ChengSheng Liao
64	22/5/2024	9:02 a.m	New composite staining	Artificial	18.78	None	Zhen Gong	ChengSheng Liao
65	22/5/2024	9:02 a.m	New composite staining	Artificial	18.75	None	Zhen Gong	ChengSheng Liao
66	22/5/2024	9:02 a.m	New composite staining	Artificial	19.11	None	Zhen Gong	ChengSheng Liao
67	22/5/2024	9:02 a.m	New composite staining	Artificial	18.78	None	Zhen Gong	ChengSheng Liao
68	22/5/2024	9:02 a.m	New composite staining	Artificial	19.24	None	Zhen Gong	ChengSheng Liao
69	22/5/2024	9:02 a.m	New composite staining	Artificial	18.89	None	Zhen Gong	ChengSheng Liao

70	22/5/2024	9:02 a.m	New composite staining	Artificial	19.04	None	Zhen Gong	ChengSheng Liao
71	22/5/2024	9:02 a.m	New composite staining	Artificial	19.31	None	Zhen Gong	ChengSheng Liao
72	22/5/2024	9:02 a.m	New composite staining	Artificial	18.74	None	Zhen Gong	ChengSheng Liao
73	22/5/2024	9:02 a.m	New composite staining	Artificial	19.01	None	Zhen Gong	ChengSheng Liao
74	22/5/2024	9:02 a.m	New composite staining	Artificial	19.35	None	Zhen Gong	ChengSheng Liao
75	22/5/2024	9:02 a.m	New composite staining	Artificial	19.35	None	Zhen Gong	ChengSheng Liao
76	22/5/2024	9:02 a.m	New composite staining	Artificial	19.31	None	Zhen Gong	ChengSheng Liao
77	22/5/2024	9:02 a.m	New composite staining	Artificial	19.18	None	Zhen Gong	ChengSheng Liao
78	22/5/2024	9:02 a.m	New composite staining	Artificial	18.67	None	Zhen Gong	ChengSheng Liao
79	22/5/2024	9:02 a.m	New composite staining	Artificial	18.68	None	Zhen Gong	ChengSheng Liao
80	22/5/2024	9:02 a.m	New composite staining	Artificial	19.26	None	Zhen Gong	ChengSheng Liao
81	22/5/2024	9:02 a.m	New composite staining	Artificial	18.84	None	Zhen Gong	ChengSheng Liao
82	22/5/2024	9:02 a.m	New composite staining	Artificial	19.31	None	Zhen Gong	ChengSheng Liao
83	22/5/2024	9:02 a.m	New composite staining	Artificial	18.81	None	Zhen Gong	ChengSheng Liao
84	22/5/2024	9:02 a.m	New composite staining	Artificial	18.93	None	Zhen Gong	ChengSheng Liao
85	22/5/2024	9:02 a.m	New composite staining	Artificial	18.7	None	Zhen Gong	ChengSheng Liao
86	22/5/2024	9:02 a.m	New composite staining	Artificial	18.69	None	Zhen Gong	ChengSheng Liao
87	22/5/2024	9:02 a.m	New composite staining	Artificial	18.75	None	Zhen Gong	ChengSheng Liao
88	22/5/2024	9:02 a.m	New composite staining	Artificial	18.77	None	Zhen Gong	ChengSheng Liao
89	22/5/2024	9:02 a.m	New composite staining	Artificial	18.74	None	Zhen Gong	ChengSheng Liao
90	22/5/2024	9:02 a.m	New composite staining	Artificial	18.84	None	Zhen Gong	ChengSheng Liao
91	22/5/2024	9:02 a.m	New composite staining	Artificial	18.65	None	Zhen Gong	ChengSheng Liao
92	22/5/2024	9:02 a.m	New composite staining	Artificial	18.67	None	Zhen Gong	ChengSheng Liao
93	22/5/2024	9:02 a.m	New composite staining	Artificial	18.75	None	Zhen Gong	ChengSheng Liao
94	22/5/2024	9:02 a.m	New composite staining	Artificial	18.72	None	Zhen Gong	ChengSheng Liao
95	22/5/2024	9:02 a.m	New composite staining	Artificial	18.9	None	Zhen Gong	ChengSheng Liao
96	22/5/2024	9:02 a.m	New composite staining	Artificial	19.21	None	Zhen Gong	ChengSheng Liao
97	22/5/2024	9:02 a.m	New composite staining	Artificial	19.12	None	Zhen Gong	ChengSheng Liao
98	22/5/2024	9:02 a.m	New composite staining	Artificial	19.19	None	Zhen Gong	ChengSheng Liao
99	22/5/2024	9:02 a.m	New composite staining	Artificial	18.92	None	Zhen Gong	ChengSheng Liao
100	22/5/2024	9:02 a.m	New composite staining	Artificial	19.08	None	Zhen Gong	ChengSheng Liao

Cell slide IOD scan data								
Numbered	Date	Time	Type of staining	Type of task	IOD	Abnormal condition	Recorder	Reviewer
1	22/5/2024	3:05 p.m	New composite staining	Single-task	17.89	None	Zhen Gong	ChengSheng Liao
2	22/5/2024	3:05 p.m	New composite staining	Single-task	18.11	None	Zhen Gong	ChengSheng Liao
3	22/5/2024	3:05 p.m	New composite staining	Single-task	17.88	None	Zhen Gong	ChengSheng Liao
4	22/5/2024	3:05 p.m	New composite staining	Single-task	17.76	None	Zhen Gong	ChengSheng Liao
5	22/5/2024	3:05 p.m	New composite staining	Single-task	18.12	None	Zhen Gong	ChengSheng Liao
6	22/5/2024	3:05 p.m	New composite staining	Single-task	18.14	None	Zhen Gong	ChengSheng Liao
7	22/5/2024	3:05 p.m	New composite staining	Single-task	17.75	None	Zhen Gong	ChengSheng Liao
8	22/5/2024	3:05 p.m	New composite staining	Single-task	18.02	None	Zhen Gong	ChengSheng Liao
9	22/5/2024	3:05 p.m	New composite staining	Single-task	18.11	None	Zhen Gong	ChengSheng Liao
10	22/5/2024	3:05 p.m	New composite staining	Single-task	17.83	None	Zhen Gong	ChengSheng Liao
11	22/5/2024	3:05 p.m	New composite staining	Single-task	18.08	None	Zhen Gong	ChengSheng Liao
12	22/5/2024	3:05 p.m	New composite staining	Single-task	17.85	None	Zhen Gong	ChengSheng Liao
13	22/5/2024	3:05 p.m	New composite staining	Single-task	17.75	None	Zhen Gong	ChengSheng Liao
14	22/5/2024	3:05 p.m	New composite staining	Single-task	17.69	None	Zhen Gong	ChengSheng Liao
15	22/5/2024	3:05 p.m	New composite staining	Single-task	17.71	None	Zhen Gong	ChengSheng Liao
16	22/5/2024	3:05 p.m	New composite staining	Single-task	17.87	None	Zhen Gong	ChengSheng Liao
17	22/5/2024	3:05 p.m	New composite staining	Single-task	17.85	None	Zhen Gong	ChengSheng Liao
18	22/5/2024	3:05 p.m	New composite staining	Single-task	17.85	None	Zhen Gong	ChengSheng Liao
19	22/5/2024	3:05 p.m	New composite staining	Single-task	17.97	None	Zhen Gong	ChengSheng Liao
20	22/5/2024	3:05 p.m	New composite staining	Single-task	18.09	None	Zhen Gong	ChengSheng Liao
21	22/5/2024	3:05 p.m	New composite staining	Single-task	18.08	None	Zhen Gong	ChengSheng Liao
22	22/5/2024	3:05 p.m	New composite staining	Single-task	17.86	None	Zhen Gong	ChengSheng Liao
23	22/5/2024	3:05 p.m	New composite staining	Single-task	17.87	None	Zhen Gong	ChengSheng Liao
24	22/5/2024	3:05 p.m	New composite staining	Single-task	17.69	None	Zhen Gong	ChengSheng Liao
25	22/5/2024	3:05 p.m	New composite staining	Single-task	17.82	None	Zhen Gong	ChengSheng Liao
26	22/5/2024	3:05 p.m	New composite staining	Single-task	17.95	None	Zhen Gong	ChengSheng Liao
27	22/5/2024	3:05 p.m	New composite staining	Single-task	18.05	None	Zhen Gong	ChengSheng Liao
28	22/5/2024	3:05 p.m	New composite staining	Single-task	18	None	Zhen Gong	ChengSheng Liao
29	22/5/2024	3:05 p.m	New composite staining	Single-task	18.13	None	Zhen Gong	ChengSheng Liao
30	22/5/2024	3:05 p.m	New composite staining	Single-task	17.69	None	Zhen Gong	ChengSheng Liao
31	22/5/2024	3:05 p.m	New composite staining	Single-task	18.02	None	Zhen Gong	ChengSheng Liao
32	22/5/2024	3:05 p.m	New composite staining	Single-task	18.11	None	Zhen Gong	ChengSheng Liao
33	22/5/2024	3:05 p.m	New composite staining	Single-task	18.02	None	Zhen Gong	ChengSheng Liao
34	22/5/2024	3:05 p.m	New composite staining	Single-task	17.65	None	Zhen Gong	ChengSheng Liao
35	22/5/2024	3:05 p.m	New composite staining	Single-task	18	None	Zhen Gong	ChengSheng Liao
36	22/5/2024	3:05 p.m	New composite staining	Single-task	17.78	None	Zhen Gong	ChengSheng Liao
37	22/5/2024	3:05 p.m	New composite staining	Single-task	17.67	None	Zhen Gong	ChengSheng Liao
38	22/5/2024	3:05 p.m	New composite staining	Single-task	17.88	None	Zhen Gong	ChengSheng Liao
39	22/5/2024	3:05 p.m	New composite staining	Single-task	17.89	None	Zhen Gong	ChengSheng Liao
40	22/5/2024	3:05 p.m	New composite staining	Single-task	17.66	None	Zhen Gong	ChengSheng Liao
41	22/5/2024	3:05 p.m	New composite staining	Single-task	17.89	None	Zhen Gong	ChengSheng Liao
42	22/5/2024	3:05 p.m	New composite staining	Single-task	18.15	None	Zhen Gong	ChengSheng Liao
43	22/5/2024	3:05 p.m	New composite staining	Single-task	18.15	None	Zhen Gong	ChengSheng Liao
44	22/5/2024	3:05 p.m	New composite staining	Single-task	17.86	None	Zhen Gong	ChengSheng Liao
45	22/5/2024	3:05 p.m	New composite staining	Single-task	17.82	None	Zhen Gong	ChengSheng Liao
46	22/5/2024	3:05 p.m	New composite staining	Single-task	17.86	None	Zhen Gong	ChengSheng Liao
47	22/5/2024	3:05 p.m	New composite staining	Single-task	17.97	None	Zhen Gong	ChengSheng Liao
48	22/5/2024	3:05 p.m	New composite staining	Single-task	18.13	None	Zhen Gong	ChengSheng Liao
49	22/5/2024	3:05 p.m	New composite staining	Single-task	17.79	None	Zhen Gong	ChengSheng Liao
50	22/5/2024	3:05 p.m	New composite staining	Single-task	17.73	None	Zhen Gong	ChengSheng Liao
51	23/5/2024	9:06 a.m	New composite staining	Single-task	17.68	None	Zhen Gong	ChengSheng Liao
52	23/5/2024	9:06 a.m	New composite staining	Single-task	17.81	None	Zhen Gong	ChengSheng Liao
53	23/5/2024	9:06 a.m	New composite staining	Single-task	17.87	None	Zhen Gong	ChengSheng Liao
54	23/5/2024	9:06 a.m	New composite staining	Single-task	17.98	None	Zhen Gong	ChengSheng Liao
55	23/5/2024	9:06 a.m	New composite staining	Single-task	18.13	None	Zhen Gong	ChengSheng Liao
56	23/5/2024	9:06 a.m	New composite staining	Single-task	17.99	None	Zhen Gong	ChengSheng Liao
57	23/5/2024	9:06 a.m	New composite staining	Single-task	17.75	None	Zhen Gong	ChengSheng Liao
58	23/5/2024	9:06 a.m	New composite staining	Single-task	18.13	None	Zhen Gong	ChengSheng Liao
59	23/5/2024	9:06 a.m	New composite staining	Single-task	17.98	None	Zhen Gong	ChengSheng Liao
60	23/5/2024	9:06 a.m	New composite staining	Single-task	18.04	None	Zhen Gong	ChengSheng Liao
61	23/5/2024	9:06 a.m	New composite staining	Single-task	17.65	None	Zhen Gong	ChengSheng Liao
62	23/5/2024	9:06 a.m	New composite staining	Single-task	17.77	None	Zhen Gong	ChengSheng Liao
63	23/5/2024	9:06 a.m	New composite staining	Single-task	17.83	None	Zhen Gong	ChengSheng Liao
64	23/5/2024	9:06 a.m	New composite staining	Single-task	17.89	None	Zhen Gong	ChengSheng Liao
65	23/5/2024	9:06 a.m	New composite staining	Single-task	17.91	None	Zhen Gong	ChengSheng Liao
66	23/5/2024	9:06 a.m	New composite staining	Single-task	17.68	None	Zhen Gong	ChengSheng Liao
67	23/5/2024	9:06 a.m	New composite staining	Single-task	17.86	None	Zhen Gong	ChengSheng Liao
68	23/5/2024	9:06 a.m	New composite staining	Single-task	17.88	None	Zhen Gong	ChengSheng Liao
69	23/5/2024	9:06 a.m	New composite staining	Single-task	17.99	None	Zhen Gong	ChengSheng Liao

70	23/5/2024	9:06 a.m	New composite staining	Single-task	18.13	None	Zhen Gong	ChengSheng Liao
71	23/5/2024	9:06 a.m	New composite staining	Single-task	18.11	None	Zhen Gong	ChengSheng Liao
72	23/5/2024	9:06 a.m	New composite staining	Single-task	18.15	None	Zhen Gong	ChengSheng Liao
73	23/5/2024	9:06 a.m	New composite staining	Single-task	17.91	None	Zhen Gong	ChengSheng Liao
74	23/5/2024	9:06 a.m	New composite staining	Single-task	18.14	None	Zhen Gong	ChengSheng Liao
75	23/5/2024	9:06 a.m	New composite staining	Single-task	18.06	None	Zhen Gong	ChengSheng Liao
76	23/5/2024	9:06 a.m	New composite staining	Single-task	18.07	None	Zhen Gong	ChengSheng Liao
77	23/5/2024	9:06 a.m	New composite staining	Single-task	18.09	None	Zhen Gong	ChengSheng Liao
78	23/5/2024	9:06 a.m	New composite staining	Single-task	17.96	None	Zhen Gong	ChengSheng Liao
79	23/5/2024	9:06 a.m	New composite staining	Single-task	18.1	None	Zhen Gong	ChengSheng Liao
80	23/5/2024	9:06 a.m	New composite staining	Single-task	17.99	None	Zhen Gong	ChengSheng Liao
81	23/5/2024	9:06 a.m	New composite staining	Single-task	18.12	None	Zhen Gong	ChengSheng Liao
82	23/5/2024	9:06 a.m	New composite staining	Single-task	17.96	None	Zhen Gong	ChengSheng Liao
83	23/5/2024	9:06 a.m	New composite staining	Single-task	17.79	None	Zhen Gong	ChengSheng Liao
84	23/5/2024	9:06 a.m	New composite staining	Single-task	17.87	None	Zhen Gong	ChengSheng Liao
85	23/5/2024	9:06 a.m	New composite staining	Single-task	17.78	None	Zhen Gong	ChengSheng Liao
86	23/5/2024	9:06 a.m	New composite staining	Single-task	17.86	None	Zhen Gong	ChengSheng Liao
87	23/5/2024	9:06 a.m	New composite staining	Single-task	17.91	None	Zhen Gong	ChengSheng Liao
88	23/5/2024	9:06 a.m	New composite staining	Single-task	17.89	None	Zhen Gong	ChengSheng Liao
89	23/5/2024	9:06 a.m	New composite staining	Single-task	18.11	None	Zhen Gong	ChengSheng Liao
90	23/5/2024	9:06 a.m	New composite staining	Single-task	17.96	None	Zhen Gong	ChengSheng Liao
91	23/5/2024	9:06 a.m	New composite staining	Single-task	17.94	None	Zhen Gong	ChengSheng Liao
92	23/5/2024	9:06 a.m	New composite staining	Single-task	18.07	None	Zhen Gong	ChengSheng Liao
93	23/5/2024	9:06 a.m	New composite staining	Single-task	17.94	None	Zhen Gong	ChengSheng Liao
94	23/5/2024	9:06 a.m	New composite staining	Single-task	18.01	None	Zhen Gong	ChengSheng Liao
95	23/5/2024	9:06 a.m	New composite staining	Single-task	17.82	None	Zhen Gong	ChengSheng Liao
96	23/5/2024	9:06 a.m	New composite staining	Single-task	17.78	None	Zhen Gong	ChengSheng Liao
97	23/5/2024	9:06 a.m	New composite staining	Single-task	17.99	None	Zhen Gong	ChengSheng Liao
98	23/5/2024	9:06 a.m	New composite staining	Single-task	17.96	None	Zhen Gong	ChengSheng Liao
99	23/5/2024	9:06 a.m	New composite staining	Single-task	18.15	None	Zhen Gong	ChengSheng Liao
100	23/5/2024	9:06 a.m	New composite staining	Single-task	17.83	None	Zhen Gong	ChengSheng Liao

Cell slide IOD scan data								
Numbered	Date	Time	Type of staining	Type of task	IOD	Abnormal condition	Recorder	Reviewer
1	23/5/2024	3:06 p.m	New composite staining	Multitask	18.07	None	Zhen Gong	ChengSheng Liao
2	23/5/2024	3:06 p.m	New composite staining	Multitask	18.29	None	Zhen Gong	ChengSheng Liao
3	23/5/2024	3:06 p.m	New composite staining	Multitask	18.5	None	Zhen Gong	ChengSheng Liao
4	23/5/2024	3:06 p.m	New composite staining	Multitask	18.42	None	Zhen Gong	ChengSheng Liao
5	23/5/2024	3:06 p.m	New composite staining	Multitask	18.54	None	Zhen Gong	ChengSheng Liao
6	23/5/2024	3:06 p.m	New composite staining	Multitask	18.1	None	Zhen Gong	ChengSheng Liao
7	23/5/2024	3:06 p.m	New composite staining	Multitask	18.45	None	Zhen Gong	ChengSheng Liao
8	23/5/2024	3:06 p.m	New composite staining	Multitask	18.53	None	Zhen Gong	ChengSheng Liao
9	23/5/2024	3:06 p.m	New composite staining	Multitask	18.19	None	Zhen Gong	ChengSheng Liao
10	23/5/2024	3:06 p.m	New composite staining	Multitask	18.17	None	Zhen Gong	ChengSheng Liao
11	23/5/2024	3:06 p.m	New composite staining	Multitask	18.41	None	Zhen Gong	ChengSheng Liao
12	23/5/2024	3:06 p.m	New composite staining	Multitask	18.05	None	Zhen Gong	ChengSheng Liao
13	23/5/2024	3:06 p.m	New composite staining	Multitask	18.17	None	Zhen Gong	ChengSheng Liao
14	23/5/2024	3:06 p.m	New composite staining	Multitask	18.28	None	Zhen Gong	ChengSheng Liao
15	23/5/2024	3:06 p.m	New composite staining	Multitask	18.06	None	Zhen Gong	ChengSheng Liao
16	23/5/2024	3:06 p.m	New composite staining	Multitask	18.38	None	Zhen Gong	ChengSheng Liao
17	23/5/2024	3:06 p.m	New composite staining	Multitask	18.38	None	Zhen Gong	ChengSheng Liao
18	23/5/2024	3:06 p.m	New composite staining	Multitask	18.26	None	Zhen Gong	ChengSheng Liao
19	23/5/2024	3:06 p.m	New composite staining	Multitask	18.45	None	Zhen Gong	ChengSheng Liao
20	23/5/2024	3:06 p.m	New composite staining	Multitask	18.42	None	Zhen Gong	ChengSheng Liao
21	23/5/2024	3:06 p.m	New composite staining	Multitask	18.53	None	Zhen Gong	ChengSheng Liao
22	23/5/2024	3:06 p.m	New composite staining	Multitask	18.11	None	Zhen Gong	ChengSheng Liao
23	23/5/2024	3:06 p.m	New composite staining	Multitask	18.41	None	Zhen Gong	ChengSheng Liao
24	23/5/2024	3:06 p.m	New composite staining	Multitask	18.41	None	Zhen Gong	ChengSheng Liao
25	23/5/2024	3:06 p.m	New composite staining	Multitask	18.14	None	Zhen Gong	ChengSheng Liao
26	23/5/2024	3:06 p.m	New composite staining	Multitask	18.18	None	Zhen Gong	ChengSheng Liao
27	23/5/2024	3:06 p.m	New composite staining	Multitask	18.36	None	Zhen Gong	ChengSheng Liao
28	23/5/2024	3:06 p.m	New composite staining	Multitask	18.31	None	Zhen Gong	ChengSheng Liao
29	23/5/2024	3:06 p.m	New composite staining	Multitask	18.53	None	Zhen Gong	ChengSheng Liao
30	23/5/2024	3:06 p.m	New composite staining	Multitask	18.26	None	Zhen Gong	ChengSheng Liao
31	23/5/2024	3:06 p.m	New composite staining	Multitask	18.08	None	Zhen Gong	ChengSheng Liao
32	23/5/2024	3:06 p.m	New composite staining	Multitask	18.35	None	Zhen Gong	ChengSheng Liao
33	23/5/2024	3:06 p.m	New composite staining	Multitask	18.45	None	Zhen Gong	ChengSheng Liao
34	23/5/2024	3:06 p.m	New composite staining	Multitask	18.44	None	Zhen Gong	ChengSheng Liao
35	23/5/2024	3:06 p.m	New composite staining	Multitask	18.08	None	Zhen Gong	ChengSheng Liao
36	23/5/2024	3:06 p.m	New composite staining	Multitask	18.51	None	Zhen Gong	ChengSheng Liao
37	23/5/2024	3:06 p.m	New composite staining	Multitask	18.28	None	Zhen Gong	ChengSheng Liao
38	23/5/2024	3:06 p.m	New composite staining	Multitask	18.31	None	Zhen Gong	ChengSheng Liao
39	23/5/2024	3:06 p.m	New composite staining	Multitask	18.46	None	Zhen Gong	ChengSheng Liao
40	23/5/2024	3:06 p.m	New composite staining	Multitask	18.23	None	Zhen Gong	ChengSheng Liao
41	23/5/2024	3:06 p.m	New composite staining	Multitask	18.53	None	Zhen Gong	ChengSheng Liao
42	23/5/2024	3:06 p.m	New composite staining	Multitask	18.35	None	Zhen Gong	ChengSheng Liao
43	23/5/2024	3:06 p.m	New composite staining	Multitask	18.09	None	Zhen Gong	ChengSheng Liao
44	23/5/2024	3:06 p.m	New composite staining	Multitask	18.45	None	Zhen Gong	ChengSheng Liao
45	23/5/2024	3:06 p.m	New composite staining	Multitask	18.24	None	Zhen Gong	ChengSheng Liao
46	23/5/2024	3:06 p.m	New composite staining	Multitask	18.1	None	Zhen Gong	ChengSheng Liao
47	23/5/2024	3:06 p.m	New composite staining	Multitask	18.11	None	Zhen Gong	ChengSheng Liao
48	23/5/2024	3:06 p.m	New composite staining	Multitask	18.41	None	Zhen Gong	ChengSheng Liao
49	23/5/2024	3:06 p.m	New composite staining	Multitask	18.32	None	Zhen Gong	ChengSheng Liao
50	23/5/2024	3:06 p.m	New composite staining	Multitask	18.29	None	Zhen Gong	ChengSheng Liao
51	24/5/2024	9:10 a.m	New composite staining	Multitask	18.16	None	Zhen Gong	ChengSheng Liao
52	24/5/2024	9:10 a.m	New composite staining	Multitask	18.31	None	Zhen Gong	ChengSheng Liao
53	24/5/2024	9:10 a.m	New composite staining	Multitask	18.29	None	Zhen Gong	ChengSheng Liao
54	24/5/2024	9:10 a.m	New composite staining	Multitask	18.53	None	Zhen Gong	ChengSheng Liao
55	24/5/2024	9:10 a.m	New composite staining	Multitask	18.33	None	Zhen Gong	ChengSheng Liao
56	24/5/2024	9:10 a.m	New composite staining	Multitask	18.16	None	Zhen Gong	ChengSheng Liao
57	24/5/2024	9:10 a.m	New composite staining	Multitask	18.35	None	Zhen Gong	ChengSheng Liao
58	24/5/2024	9:10 a.m	New composite staining	Multitask	18.4	None	Zhen Gong	ChengSheng Liao
59	24/5/2024	9:10 a.m	New composite staining	Multitask	18.26	None	Zhen Gong	ChengSheng Liao
60	24/5/2024	9:10 a.m	New composite staining	Multitask	18.54	None	Zhen Gong	ChengSheng Liao
61	24/5/2024	9:10 a.m	New composite staining	Multitask	18.28	None	Zhen Gong	ChengSheng Liao
62	24/5/2024	9:10 a.m	New composite staining	Multitask	18.08	None	Zhen Gong	ChengSheng Liao
63	24/5/2024	9:10 a.m	New composite staining	Multitask	18.1	None	Zhen Gong	ChengSheng Liao
64	24/5/2024	9:10 a.m	New composite staining	Multitask	18.34	None	Zhen Gong	ChengSheng Liao
65	24/5/2024	9:10 a.m	New composite staining	Multitask	18.12	None	Zhen Gong	ChengSheng Liao
66	24/5/2024	9:10 a.m	New composite staining	Multitask	18.24	None	Zhen Gong	ChengSheng Liao
67	24/5/2024	9:10 a.m	New composite staining	Multitask	18.21	None	Zhen Gong	ChengSheng Liao
68	24/5/2024	9:10 a.m	New composite staining	Multitask	18.42	None	Zhen Gong	ChengSheng Liao
69	24/5/2024	9:10 a.m	New composite staining	Multitask	18.05	None	Zhen Gong	ChengSheng Liao

70	24/5/2024	9:10 a.m	New composite staining	Multitask	18.23	None	Zhen Gong	ChengSheng Liao
71	24/5/2024	9:10 a.m	New composite staining	Multitask	18.13	None	Zhen Gong	ChengSheng Liao
72	24/5/2024	9:10 a.m	New composite staining	Multitask	18.05	None	Zhen Gong	ChengSheng Liao
73	24/5/2024	9:10 a.m	New composite staining	Multitask	18.07	None	Zhen Gong	ChengSheng Liao
74	24/5/2024	9:10 a.m	New composite staining	Multitask	18.11	None	Zhen Gong	ChengSheng Liao
75	24/5/2024	9:10 a.m	New composite staining	Multitask	18.32	None	Zhen Gong	ChengSheng Liao
76	24/5/2024	9:10 a.m	New composite staining	Multitask	18.48	None	Zhen Gong	ChengSheng Liao
77	24/5/2024	9:10 a.m	New composite staining	Multitask	18.51	None	Zhen Gong	ChengSheng Liao
78	24/5/2024	9:10 a.m	New composite staining	Multitask	18.23	None	Zhen Gong	ChengSheng Liao
79	24/5/2024	9:10 a.m	New composite staining	Multitask	18.05	None	Zhen Gong	ChengSheng Liao
80	24/5/2024	9:10 a.m	New composite staining	Multitask	18.51	None	Zhen Gong	ChengSheng Liao
81	24/5/2024	9:10 a.m	New composite staining	Multitask	18.11	None	Zhen Gong	ChengSheng Liao
82	24/5/2024	9:10 a.m	New composite staining	Multitask	18.15	None	Zhen Gong	ChengSheng Liao
83	24/5/2024	9:10 a.m	New composite staining	Multitask	18.06	None	Zhen Gong	ChengSheng Liao
84	24/5/2024	9:10 a.m	New composite staining	Multitask	18.43	None	Zhen Gong	ChengSheng Liao
85	24/5/2024	9:10 a.m	New composite staining	Multitask	18.22	None	Zhen Gong	ChengSheng Liao
86	24/5/2024	9:10 a.m	New composite staining	Multitask	18.09	None	Zhen Gong	ChengSheng Liao
87	24/5/2024	9:10 a.m	New composite staining	Multitask	18.2	None	Zhen Gong	ChengSheng Liao
88	24/5/2024	9:10 a.m	New composite staining	Multitask	18.43	None	Zhen Gong	ChengSheng Liao
89	24/5/2024	9:10 a.m	New composite staining	Multitask	18.37	None	Zhen Gong	ChengSheng Liao
90	24/5/2024	9:10 a.m	New composite staining	Multitask	18.22	None	Zhen Gong	ChengSheng Liao
91	24/5/2024	9:10 a.m	New composite staining	Multitask	18.11	None	Zhen Gong	ChengSheng Liao
92	24/5/2024	9:10 a.m	New composite staining	Multitask	18.2	None	Zhen Gong	ChengSheng Liao
93	24/5/2024	9:10 a.m	New composite staining	Multitask	18.32	None	Zhen Gong	ChengSheng Liao
94	24/5/2024	9:10 a.m	New composite staining	Multitask	18.23	None	Zhen Gong	ChengSheng Liao
95	24/5/2024	9:10 a.m	New composite staining	Multitask	18.23	None	Zhen Gong	ChengSheng Liao
96	24/5/2024	9:10 a.m	New composite staining	Multitask	18.25	None	Zhen Gong	ChengSheng Liao
97	24/5/2024	9:10 a.m	New composite staining	Multitask	18.26	None	Zhen Gong	ChengSheng Liao
98	24/5/2024	9:10 a.m	New composite staining	Multitask	18.49	None	Zhen Gong	ChengSheng Liao
99	24/5/2024	9:10 a.m	New composite staining	Multitask	18.58	None	Zhen Gong	ChengSheng Liao
100	24/5/2024	9:10 a.m	New composite staining	Multitask	18.53	None	Zhen Gong	ChengSheng Liao
